# Impact of Vertical Blender Unit Parameters on Subsequent Process Parameters and Tablet Properties in a Continuous Direct Compression Line

**DOI:** 10.3390/pharmaceutics14020278

**Published:** 2022-01-25

**Authors:** Marius J. Kreiser, Christoph Wabel, Karl G. Wagner

**Affiliations:** 1 Product and Process Development, Pfizer Manufacturing Deutschland GmbH, 79108 Freiburg, Germany; marius.kreiser@pfizer.com (M.J.K.); christoph.wabel@pfizer.com (C.W.); 2 Department of Pharmaceutical Technology and Biopharmaceutics, University of Bonn, 53121 Bonn, Germany

**Keywords:** continuous manufacturing, continuous mixing technology, vertical blender, direct compression, lubrication, material characterization

## Abstract

The continuous manufacturing of solid oral-dosage forms represents an emerging technology among the pharmaceutical industry, where several process steps are combined in one production line. As all mixture components, including the lubricant (magnesium stearate), are passing simultaneously through one blender, an impact on the subsequent process steps and critical product properties, such as content uniformity and tablet tensile strength, is to be expected. A design of experiment (DoE) was performed to investigate the impact of the blender variables hold-up mass (HUM), impeller speed (IMP) and throughput (THR) on the mixing step and the subsequent continuous manufacturing process steps. Significant impacts on the mixing parameters (exit valve opening width (EV), exit valve opening width standard deviation (EV SD), torque of lower impeller (T_L_), torque of lower impeller SD (T_L_ SD), HUM SD and blend potency SD), material attributes of the blend (conditioned bulk density (CBD), flow rate index (FRI) and particle size (d_10_ values)), tableting parameters (fill depth (FD), bottom main compression height (BCH) and ejection force (EF)) and tablet properties (tablet thickness (TT), tablet weight (TW) and tensile strength (TS)) could be found. Furthermore, relations between these process parameters were evaluated to define which process states were caused by which input variables. For example, the mixing parameters were mainly impacted by impeller speed, and material attributes, FD and TS were mainly influenced by variations in total blade passes (TBP). The current work presents a rational methodology to minimize process variability based on the main blender variables hold-up mass, impeller speed and throughput. Moreover, the results facilitated a knowledge-based optimization of the process parameters for optimum product properties.

## 1. Introduction

**Figure 1:**
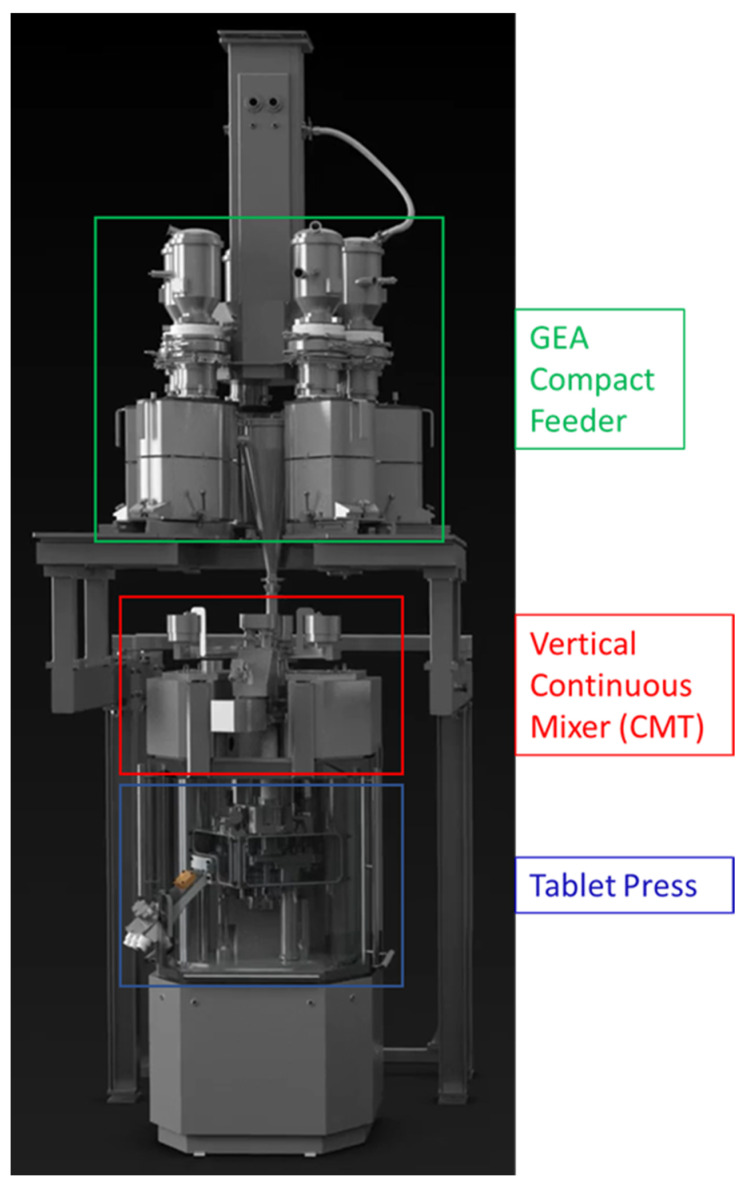
Overview of the direct compression line used for this trial.

Continuous manufacturing lines are supplied by various vendors and distributed amongst the pharmaceutical industry. Next to the benefit of continuous manufacturing, the modular setup allows for an easier transfer amongst various production sites, as the setup can be more easily cloned from the pilot plant or launch site [1]. The PCMM (portable, continuous, modular and miniature) installed at the Pfizer site in Freiburg, Germany, consists of a GEA Compact Feeder, a vertical continuous blender (CMT—continuous mixing technology), a MODUL™ P tablet press equipped with an NIR (near infrared) probe installed in the feed frame and an at-line combi-tester to analyze tablet properties, such as thickness, weight and crushing strength (Figure 1).

Basically, for each raw material, the powder is transferred from a polyethylene bag via a top-up valve into an agitated hopper, where co-rotating screws supply the powder by the loss in weight (LiW) principle at a composition related feed rate. The continuous process demands a periodical refill of the hopper triggered by a defined refill level, performed by a rotating volumetric refill device with flexible volume inserts [2,3].

Since feeding is the first step within a continuous process, it is consequently one of the first critical control elements besides the material attributes. Accurate feeding is substantial for the quality of a continuous process to avoid deviations regarding the quality of blend and content uniformity of the tablets [4,5,6,7,8]. 

**Figure 2:**
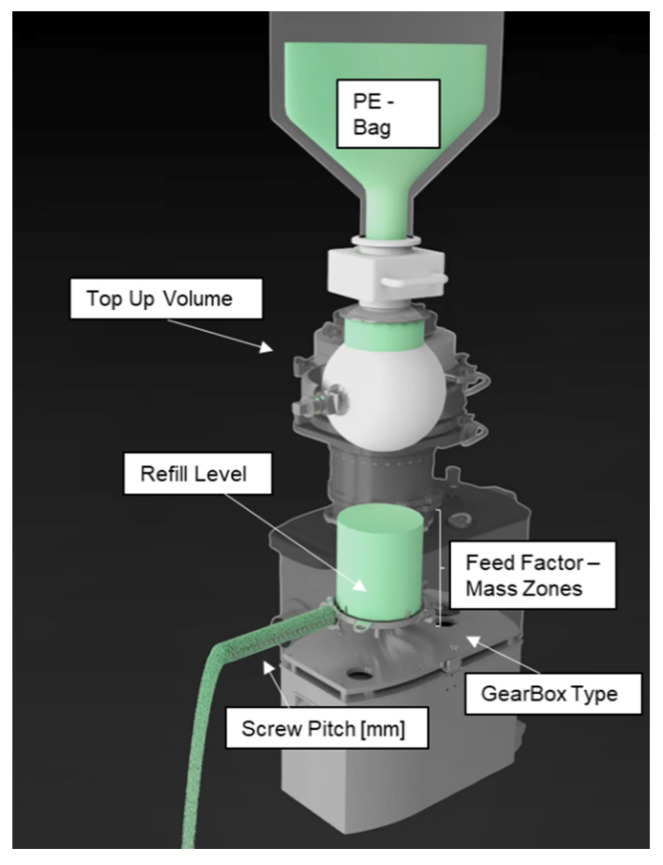
Overview of a GEA Compact Feeder and corresponding adjustment options.

To provide low variability in feed rate, the optimal feeder design and the corresponding parameter settings, such as refill level, top-up volume, screw pitch, feed-factor array (governing dosing in volumetric mode during e.g., refill) and gearbox type (Figure 2), should be individually adjusted based on composition, throughput and powder attributes [3,4,7,8,9,10,11,12,13].

**Figure 3:**
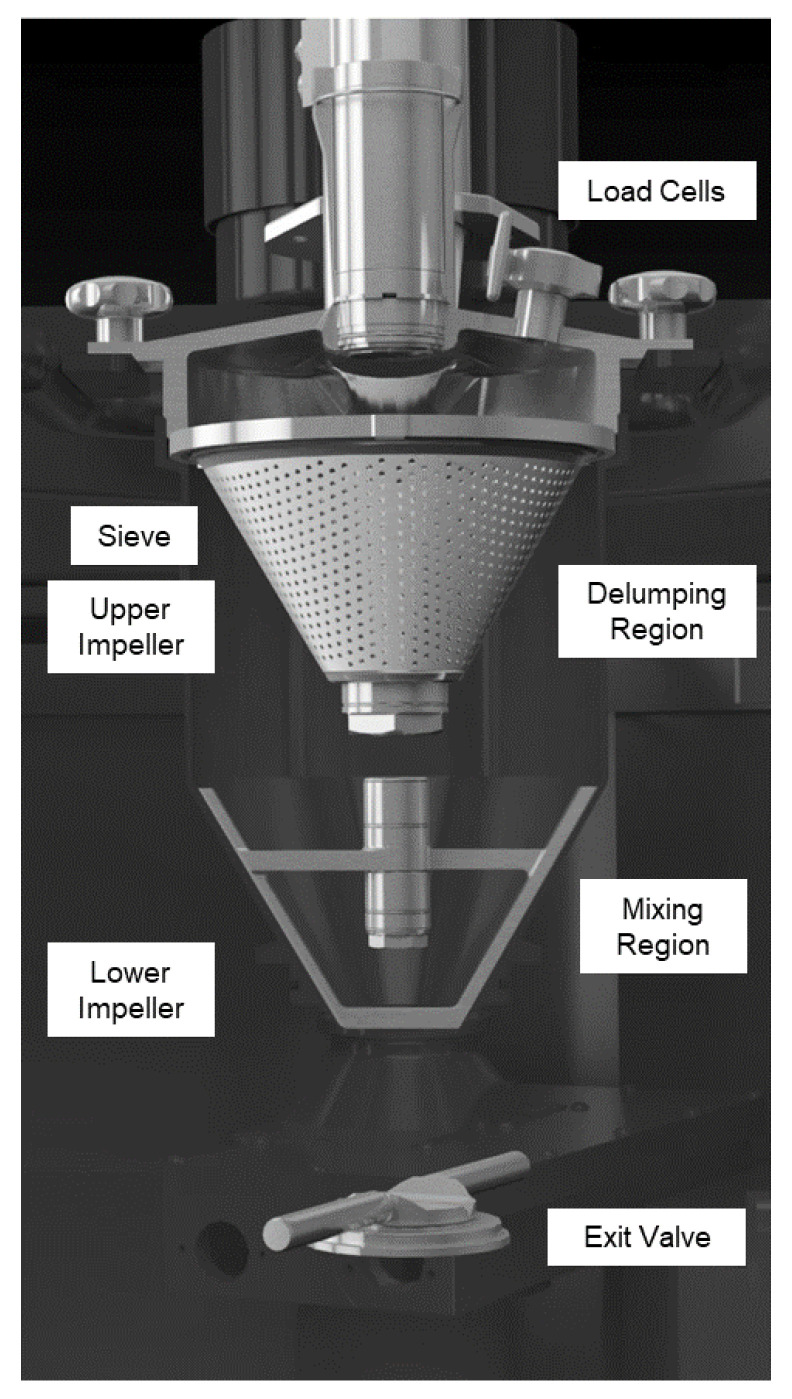
Overview of the CMT. View of the upper impeller is obstructed by the sieve.

Several feeders supply each raw material separately, and the powder falls through the conical-shaped inlet hopper into the vertical continuous mixer. It is composed of two regions: the upper delumping region and the lower mixing region (Figure 3). In both, the impellers can be adjusted independently regarding speed, direction and vertical position, i.e., the gap between impeller and conical sieve. In the delumping region, a downstream sieve (d = 2.1 mm) is set to delump possible agglomerates. The powder leaves the upper region and arrives in the conical mixing region, where a second impeller is mounted. The whole setup of the CMT is attached to load cells, which monitor the weight of the powder within the mixer. This hold-up mass (HUM) is defined in the recipe and determines the mass, will always be mixed in the CMT continuously throughout the process. 

Other papers focus on a horizontal continuous mixer, where the HUM is considered a function of flow rate and impeller speed and cannot be set individually [14,15]. In contrast, the HUM in a vertical continuous mixer remains constant, and various shear rates (impeller speeds) can be applied despite a constant residence-time distribution (RTD) [8]. As in this case the continuous DC line includes only one mixing step for all mixture components, including the lubricant. An impact especially on lubricant-sensitive mixtures, as well as on the blend uniformity of the mixture and, subsequently, content uniformity of the tablets, can be expected [16,17,18].

The exit valve is located at the bottom of the CMT. By means of a proportional–integral–derivative (PID) control loop, the exit valve opening width is adjusted automatically based on the current HUM value in order to keep the mass of the CMT constant. The controlled exit valve ensures that the same amount of mass entering the CMT will simultaneously leave the CMT (mass_in_ = mass_out_). Feed fluctuations of each feeder and the respective variability in the mass flow can be balanced that way. Smaller exit valve opening widths are recommended so that newly entering raw materials can be properly mixed together with the blend that is already present in the blender. Otherwise, unmixed or poorly mixed material can pass by and leave the CMT without being blended, causing content-uniformity variability [8].

The mean residence time (MRT, Equation (1)) of a particle can be calculated based on the overall throughput and the HUM. It reflects the mixing period of that particle within the CMT [8].

(1) $$\mathrm{MRT}\;\left[\min\right]=\frac{\mathrm{HUM}\;\left[\mathrm{kg}\right]}{\mathrm{THR}\;\left[\frac{\mathrm{kg}}{h}\right]}*60\frac{\min}{h}$$

The total blade passes (TBP, Equation (2)) reveals how often the impeller, on average, will pass a particle and show the intensity of the shear transmitted to the powder. With an increasing number of revolutions and respectively increased shear, a lubricant, such as magnesium stearate (MgSt), can be introduced more homogeneously into the blend or even filmed onto the particles, potentially resulting in decreasing tensile strength of tablets. Therefore, particular attention is paid to the single mixing step in the CMT, where the lubricant will be mixed right from the start, together with the remaining raw materials, potentially resulting in a narrow process window between a homogeneous and an over-lubricated blend. Hence, it is required to set a suitable combination for HUM and IMP to ensure that TS and disintegration, as well as dissolution time, are within specification [19,20,21,22,23,24,25]. Thresholds regarding HUM and IMP are, besides the mass balance model (MBM), part of the control strategy of the CMT. If the process values exceed the specific limits, an alarm occurs and the process stops. Furthermore, variations in HUM and IMP could also impact the exit valve opening width and, therefore, the mixing quality.

(2) TBP=MRT [min]∗IMP [rpm]

After the powder exits the CMT, it travels through the feed chute into the feed frame, where powder will be held up and be fed into the tablet press. Position sensors in the feed chute measure the filling levels. Using an internal feedback loop, we can control the turret speed of the tablet press according to the filling levels, thus preventing powder from backing up or the tablet press from running empty. An increasing feed-chute level results in an increased turret speed of the tablet press, i.e., increased powder demand, and vice versa. 

The NIR probe in the feed frame is the first chemometric measurement in the process. It is therefore important to understand the impact of upstream settings and process states on the conformity of potency, as predicted by the NIR model.

The NIR probe measures a defined volume of the powder. The corresponding spectra are used to predict the API (active pharmaceutical ingredient) content. If inhomogeneity of the blend or variability in the upstream process units occurs, it can consequently be detected with NIR and is seen as a disturbance in the blend potency measured by NIR inside the feed frame [26].

Depending on the chosen control strategy, the impacted tablets can be diverted into the waste channel if the signals exceed the specification limits. As soon the signals are within specification limits again, the diverter switches back to the good product channel after a defined lead-lag time [27,28,29].

The tablet press was running with a control mode enabled, wherein the tablet weight control is based on the pre-compression displacement and the fill depth is adjusted accordingly. The bottom main compression height controls the thickness and compression force and, therefore, the crushing strength of the tablets. At the end of the tablet press, the tablets can be directed into the good channel, diverted into the waste channel or directed to the combi-tester, where at-line measurements regarding tablet properties can be performed in containment (see Section 2.7).

This paper assesses to what extent HUM, IMP and THR impact the downstream process of a direct compression mixture. It focuses on correlations and coherences and evaluates the predictability of process parameters based on the CMT settings, especially since the lubricant and all other formulation constituents are mixed simultaneously in one single mixing step. As a model formulation, Saccharin Monohydrate was used as API surrogate.

## 2. Materials and Methods

### 2.1. Materials

For this trial, saccharin sodium monohydrate (JMC, Ulsan, South Korea), microcrystalline cellulose (Avicel PH 102, FMC, Cork, Ireland), calcium di-phosphate (A-Tab, Innophos, Chicago Heights, IL, USA), sodium starch glycolate (Roquette, Lestrem, France) and magnesium stearate V (Mallinckrodt, St. Louis, MO, USA) were used. 

### 2.2. DoE Settings

A central composite face design with a star points at the face of each side defined by a 2-level factorial design was conducted by using MODDE Pro 12.1 (Satorius Stedim Data Analytics AB, Umea, Sweden) (Table 2). A quadratic model was used, wherein the following parameters (Table 1) were considered:

Compounds and composition remained constant over the entire experiment. In general, 17 runs, including 3 replicates of a center point, were performed (Table 2). After adjusting the new CMT parameters, a transition phase was initiated (3 × MRT) to wash out the powder mixed at the former setting. A compression-force profile was conducted by using 118, 157, 169, 236 and 275 MPa compression pressure for each phase. Subsequently, the process was run for at least 10 min in a steady-state phase. 

During the transition phase and the compression-force profile, the tablet press was operated in manual mode, without using the combi-tester, to analyze tablet properties. In manual mode, samples were taken and weighed manually to select the correct fill depth. During each steady-state phase, manual mode was switched to automatic mode, in which the NIR probe was active. For each steady state phase, 275 MPa compression pressure was set; a tablet sample was taken in the middle of the steady state phase, using the combi-tester; and a powder sample was withdrawn at the end of each steady state phase by opening the sampling port underneath the feed frame and collecting approximately 300 g of powder.

### 2.3. Feeder Settings

The continuous manufacturing line used was equipped with PID-controlled LiW feeders. To ensure consistent powder supply, the following feeder settings were used (Table 3).

### 2.4. Bulk and Tapped Density 

Bulk and tapped density were measured by using an Erweka SVM 222 (ERWEKA GmbH, Langen, Germany) according to Ph.Eur. A 250 mL graduated flask was used and filled with an appropriate amount of powder of each raw material and blend. The initial volume and the volume (V_0_) after 750 and 1250 taps were noted. Each sample was analyzed in triplicate. Hausner Ratio and Carr’s Index were calculated as shown in Equations (3) and (4) and interpreted as shown in Table 4.

(3) $$\mathrm{Hausner}\;\mathrm{Ratio}=\frac{\rho_{\mathrm{tapped}}}{\rho_{\mathrm{bulk}}}\;$$

(4) $$\mathrm{Carr}\;\mathrm{Index}=\frac{\rho_{\mathrm{tapped}}-\rho_{\mathrm{bulk}}}{\rho_{\mathrm{tapped}}}*100$$

### 2.5. Freeman Powder Rheometer FT4 

The FT4 Powder Rheometer (Freeman Technology Inc., Worcestershire, UK) was used to characterize flow properties of powders and granulates. For this trial, 3 methods (stability and variable flow rate, powder compressibility and shear cell) were used to analyze the impact of CMT parameters on the flowability of the resulting blends.

#### 2.5.1. Stability and Variable Flow Rate

In this trial, a cylindrical 25 mm × 25 mL split vessel was used. After an initial condition cycle, the powder was split to obtain a defined amount of powder to ensure reproducible measurements. The actual testing consisted of seven alternating conditioning and test cycles where the blade was inserted in the powder bed and was moved downward, with a rotational blade tip speed of 100 mm/s, to remove history and operator influence. Subsequently, 4 cycles with decreasing blade tip speed (100, 70, 40 and 10 mm/s) were performed. The required energy is based on the resistance of the blade to flow in the downward motion [31]. 

The basic flow energy (BFE) is defined by the required energy to move the blade downward at test-cycle 7. The specific energy (SE) represents the energy that is required during an upward traverse at the same test cycle. The stability index (SI) is calculated by the ratio of the energy at test-cycle 7 and test-cycle 1. The flow-rate index (FRI) reflects the results of the reducing blade-tip speed, where the energy of the lowest rotational speed and the highest is set in ratio.

(5) $$\mathrm{FRI}=\frac{\mathrm{energy}\;\mathrm{test}\;11\;\left(10\;\frac{\mathrm{mm}}{s}\right)}{\mathrm{energy}\;\mathrm{test}\;8\;\left(100\;\frac{\mathrm{mm}}{s}\right)}$$

Basically, at higher flow rates, less energy is required, since the entrained air acts as a lubricant. At lower flow rates, the powder in front of the blades is more likely to be consolidated, due to the absence of entrained air; therefore, the interlocking of particles is more probable. Consequently, higher FRI values are common for cohesive powders. In this study, FRI values < 1 are shown; they are typical for powders or blends containing lubricants. The conditioned bulk density (CBD) was measured after the initial conditioning cycle and the split of the powder, where agglomerates and air inclusions could be evened to ensure reproducible measurements [11,30,32,33,34].

#### 2.5.2. Powder Compressibility

The compressibility method was used to investigate how the density of the measured powder changes with increasing normal stress. A split vessel (25 mm × 10 mL) was used for this trial. After three conditioning cycles, the powder was split, and the blade was changed for a vented piston. In total, 8 compression steps were performed (1, 2, 4, 6, 8, 10, 12, and 15 kPa) and were held for 60 s at each force. In this work, only compressibility (change in volume after compression (%)) was used. Low compressibility values occurred for powders with a low amount of entrained air where particles are packed compactly. High compressibility values were seen if voids within the powder occurred. This was likely with cohesive powders [30,35]. 

#### 2.5.3. Shear Cell

A shear cell test was performed by using the FT4. For this method, a 25 mm × 10 mL split vessel was used. As normal stress, 7, 6, 5, 4 and 3 kPa were adjusted, and the initial consolidation stress was 9 kPa. 

For this method, a τ−σ-diagram can be obtained, where one pre-shear point and five yield points can be observed. Using a Mohr circle analysis, a linearized yield locus can be obtained, where the τ-axis intersection is interpreted as cohesion and presents the obtained shear stress during powder deformation when no normal stress is applied [11,36,37,38,39].

### 2.6. Particle Size Distribution

For particle size measurements, a Sympatec QicPic (Sympatec GmbH, Clausthal-Zellerfeld, Germany) was used. It is a dynamic high-speed image-analysis system with a LED pulse-light source and high-resolution high-speed camera. An M7 lens was used that covers particles between 4.2 and 2888 µm. Dispersion pressure was set to 1 bar for all raw materials and blends to maintain comparability. A dry dispersion line RODOS/L with VIBRI attachment was in place, and the sample size remained constant for each material (5 mL). To determine the particle size, the EQPC method was used, where d_10_, d_50_ and d_90_ values were obtained (see Supplementary Table S34).

### 2.7. Tableting

A MODUL™ P tablet press (GEA Pharma Systems, Courtoy™, Halle, Belgium) was implemented at the end of the continuous manufacturing line. Mode 2 (Courtoy dual-control force method) was selected, where the tablet weight control is based on pre-compression displacement measurements adjusting the fill depth, accordingly [40].

Round convex tablets with an 11 mm diameter and 1.12 mm cup height were manufactured. During steady state, a target compression pressure of 275 MPa was set.

The target tablet weight of 600 mg, tablet crushing strength and tablet thickness were tested periodically in the middle of each steady state, using the at-line combi-tester (Kraemer Elektronik GmbH, Darmstadt, Germany).

The feed chute level was controlled to a constant level at 40%, and the paddle speed remained constant at 45 and 40 rpm. Turret speed set-points and speed tolerances of the tablet press were adapted to the respective mass throughput (11 rpm ± 2.2 rpm; 21 rpm ± 4.2 rpm and 32 rpm ± 6.4 rpm).

#### Tensile Strength

The tensile strength of the convex round tablets was calculated based on the following equation [41]:

(6) $$\mathrm{Tensile}\;\mathrm{Strength}=\frac{10P_{s}}{\pi D^{2}}{\left(2.84\frac{t}{D}-0.126\frac{t}{W}+3.15\frac{W}{D}+0.01\right)}^{-1}$$

where *P_s_* = tablet core crushing strength, *D* = tablet core diameter, *t* = tablet core thickness and *W* = cylinder length. Tablet-crushing strength was measured by using the combi-tester, which is directly connected to the continuous manufacturing line.

### 2.8. Blend Potency

To analyze the impact of the CMT settings on the blend potency, an NIR spectrometer (SentroProbe DR LS NIR 170C ATEX, Sentronic GmbH, Dresden, Germany) was installed in the feed frame, with an insertion depth of 1 mm. Using PharmaMV 5.3 (Perceptive Engineering, Daresbury, UK), we recorded a spectrum every 4 s. Approximately 150–200 mg of the blend was measured during one measurement cycle. The collected data were preprocessed by first applying the Savitzky–Golay filter and then the standard normalize variate method (SNV). After that, the data were processed by a partial least square (PLS) regression model. The integration time was 9 ms, with 133 average scans. 

### 2.9. Software

#### 2.9.1. MODDE

The DoE was designed by using MODDE Pro 12.1. A multiple linear regression (MLR) model was used to evaluate the significance of the input factors on the responses. Furthermore, MODDE was used to obtain model equations to predict the responses.

#### 2.9.2. Osi Pi

A considerable benefit of the PCMM is the implementation of OsiPi (OsiSoft, San Leandro, CA, USA), which enables access to all essential process values. All data generated by the PCMM are continuously monitored and stored by using OsiPi. 

Pi Vision is a web-based tool wherein process data can be visualized in real time. Since the process data are stored in the PI Server, PiVision also can visualize previous batches if process states need to be evaluated retrospectively. For this trial, all process-related data were gathered by using PiDataLink, which is an Add-In to Excel (Microsoft Corporation, Redmond, Washington, USA) that enables data to be imported from the PI Server.

#### 2.9.3. GraphPad Prism

GraphPad Prism 9 (GraphPad Software, Inc., San Diego, CA, USA) was used to generate the figures and to calculate the correlations (Pearson correlations) between the process parameters, including the *p*-values. All correlation coefficients are shown Supplementary Figure S57. To evaluate the size of the correlation, the following thumb rule was used (Table 5): 

## 3. Results and Discussion

### 3.1. DoE Results

**Figure 4:**
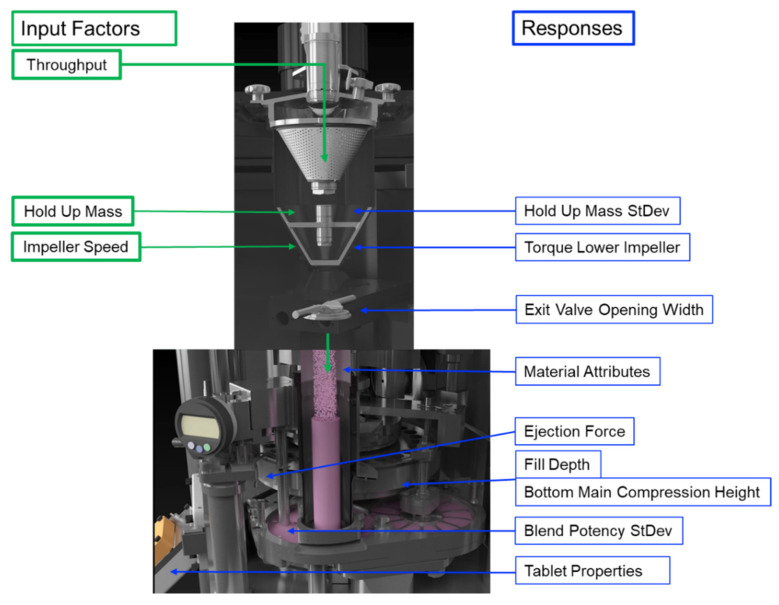
Process overview of input factors (green, **left** side) and observed responses (blue, **right** side).

The DoE reveals to what extent the input variables throughput (THR), hold-up mass (HUM) and impeller speed (IMP) affect the response parameters, such as the exit valve opening width and SD, torque of the lower impeller and corresponding SD, HUM SD and blend potency uniformity, as measured by NIR, in regard to the mixing step. Furthermore, the impact on material attributes of the blend (FRI, CBD and d_10_ values), tablet press parameters (FD, BCH and EF) and tablet properties (TS, TW, TT and corresponding standard deviation) are presented. A visualization where responses are expected is shown in Figure 4. The data were fitted by using an MLR model, wherein significant model terms are identifiable when error bars (=95% confidence interval) do not cross the zero-line. Corresponding-fit statistics are shown in Supplementary Tables S2–S33. In this paper, models with Q^2^ > 0.500 (=estimate of prediction precision) and R^2^ ≥ 0.800 (=model fit) are considered good models, indicating a significant correlation between input variables and responses.

#### 3.1.1. Mixing Parameters

**Figure 5:**
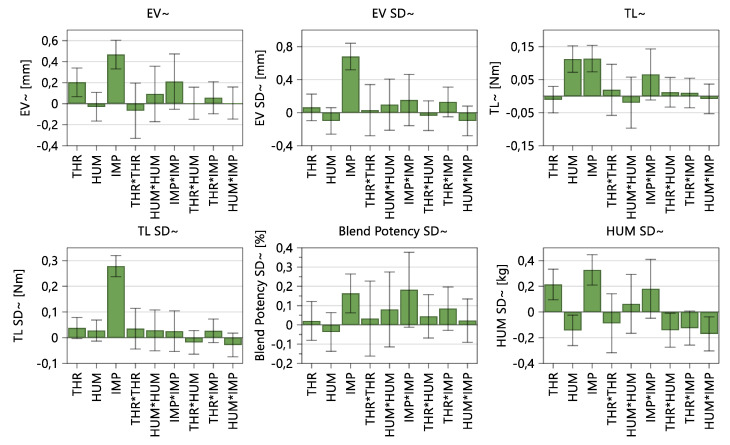
Coefficients plot of the impact of input variables on responses regarding the blending unit and uniformity of the blend. The 95% confidence interval is displayed as an error bar.

For each presented response regarding mixing quality, impeller speed is a significant model term (Figure 5). The DoE showed that the influence on the exit valve opening width is driven by THR and IMP, resulting in higher opening widths if throughput and impeller speed are high as well. Regarding variability in EV, torque and blend potency, the impeller speed is the only significant model term. For torque values, HUM and IMP seem to share the same extent of deflection. With regard to HUM SD values, all three input factors and HUM*IMP were significant.

As shown in Table 6, Q2 and R2 imply, that exit valve opening width (+SD) and torque (+SD) can be considered good models. As the variabilities of the responses were not linearly distributed a logarithmic data transformation was conducted (as shown in the corresponding chapters and Supplementary Figures S7 and S11).

Further details regarding fit statistics and model equations are shown in Supplementary Section A, “Summary of Fit: Mixing Parameter”.

#### 3.1.2. Material Attributes of the Blend

**Figure 6:**
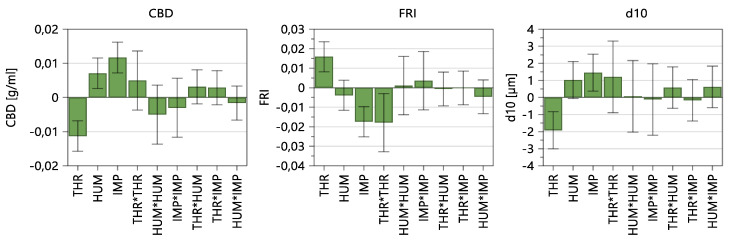
Coefficients plot of model terms regarding material attributes of the blends. The 95% confidence interval is displayed as an error bar.

The conditioned bulk density (CBD), flow-rate index (FRI) and d_10_ values of the blend were evaluated (Figure 6). THR, HUM and IMP show a similar impact on CBD and d_10_ values of the powder. In contrast, the coefficients regarding FRI show a positive impact of THR and a negative influence by IMP and THR*THR.

Table 7 shows the fit statistics after removing non-significant model terms, whereby models regarding CBD, FRI and d_10_ can be considered good models. For further details, see Supplementary Section B, “Summary of Fit: Material Attributes of the Blend”.

#### 3.1.3. Tableting Parameters

**Figure 7:**
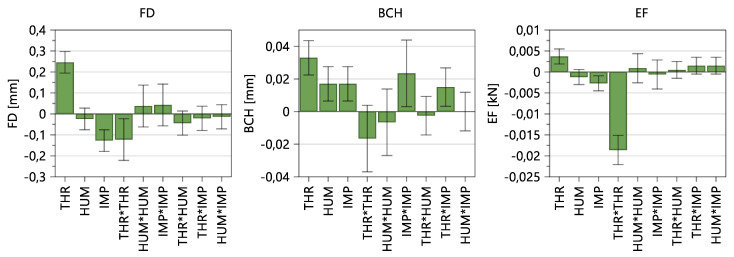
Coefficients plot of model terms regarding tablet press parameters. The 95% confidence interval is displayed as an error bar.

Regarding tableting parameters, the fill depth, bottom main compression height and ejection force were evaluated, wherein throughput and impeller speed are significant model terms for all three parameters (Figure 7). That means, these input factors have a statistically significant impact on all three tableting parameters. For example, higher throughput and lower impeller speed result in lower TBP and, therefore, in lower lubrication, leading to lower powder densities, higher required fill depths and higher ejection forces. 

Furthermore, the fill depth and ejection force share the same deflection of the three significant model terms, namely THR, IMP and THR*THR.

Table 8 shows the fit statistics after removing non-significant model terms. All three parameters show high values regarding Q^2^ and R^2^. For further information regarding fit statistics and model equations, see Supplementary Section C, “Summary of Fit: Tablet Press Parameters”.

#### 3.1.4. Tablet Properties

To investigate the impact of CMT parameters on the tablet properties, the tensile strength (TS), tablet weight (TW) and tablet thickness (TT) obtained during steady state at 275 MPa compression pressure were evaluated.

**Figure 8:**
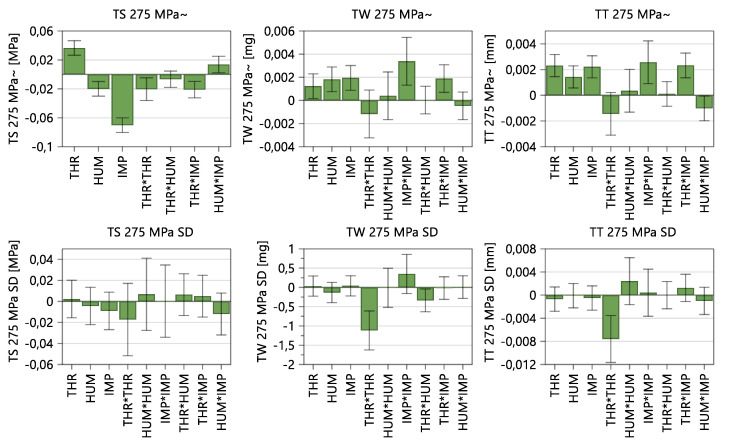
Model terms regarding tensile strength (TS), tablet weight (TW), tablet thickness (TT) and corresponding standard deviation. The 95% confidence interval is displayed as an error bar.

In Figure 8, it can be observed that, besides the three input factors, namely THR, HUM and IMP, THR*THR, THR*IMP and HUM*IMP are significant model terms for tensile strength. That means, higher THR, lower HUM and lower IMP resulted in lower TBP and, therefore, lower lubrication, which increased the tensile strength of the tablets. Further explanations regarding TBP and tablet properties can be seen in the paragraph “Tensile Strength”.

On the other hand, the tablet weight and thickness are both influenced by similar input variables. As the tablet-weight variance was always within control limits, an automatic weight adjustment did not occur. Consequently, TW was impacted by the density of the blends and FD. Considering the MLR, the high IMP, high IMP^2^ and high THR*IMP resulted in higher TBP and higher densities. Since the FD adjustments only occurred occasionally when the displacement at the pre-compression exceeded internal limits at which the calculated weights are too high/low, higher powder density resulted in higher TW. Regarding variability in tablet properties, throughput has the highest impact on tablet weight and thickness standard deviations, whereas no significant model term regarding TS SD could be found.

According to Table 9, tensile strength, tablet weight and tablet thickness can be considered good models. Again, as the variabilities of the responses were not linearly distributed, a logarithmic data transformation was conducted. Corresponding figures, model equations and fit statistics are shown in Supplementary Section D, “Summary of Fit: Tablet Properties”.

### 3.2. Response Factors

**Figure 9:**
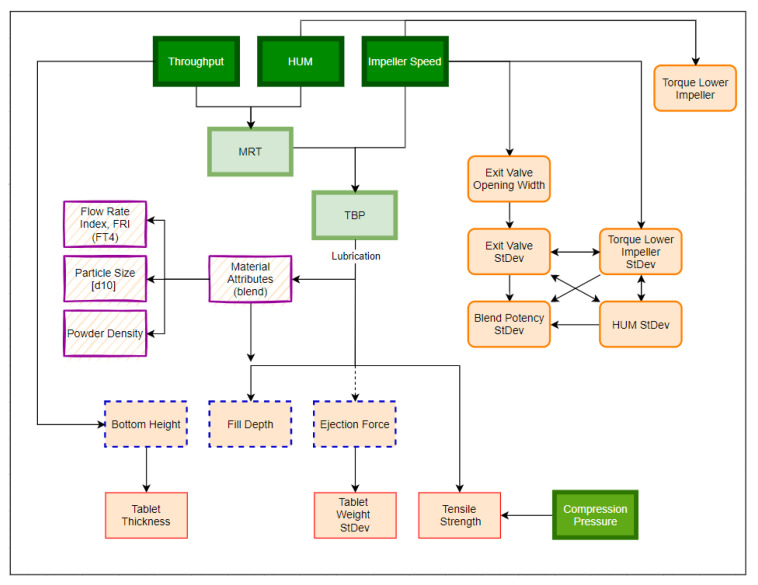
Qualitative overview of process parameter connections and correlations. Input factors are marked in dark green (thick borders), confounding input parameters are marked in light green and the considered response parameters are shown in light orange. The color/shape of the borders classifies the responses into mixing parameters (orange line, rounded corners), material attributes of the blend (purple, striped background), tableting parameters (blue, dotted borders) and tablet properties (red, thin borders). Compression pressure (green) is considered an independent input factor of the tablet press.

For recapitulation, Figure 9 demonstrates the relationships between all parameters obtained and evaluated within this DoE. Starting from the CMT settings, the flowchart depicts the downstream process parameters where correlations are expected to be found. 

#### 3.2.1. Mixing Parameters

##### Exit-Valve-Opening Width

As presented in Figure 5, the throughput and impeller speed were the significant model terms for the exit valve opening width (Q^2^ = 0.860 and R^2^ = 0.905). The low model validity observed was due to the extremely low variability seen in the replicated center points, and, hence, it is not a cause for concern.

**Figure 10:**
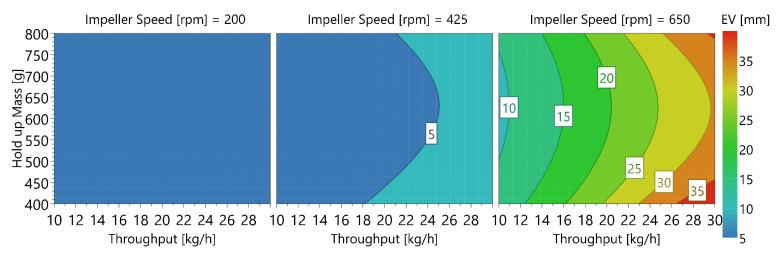
Contour plot of exit valve opening width in dependence of THR, HUM and IMP.

**Figure 11:**
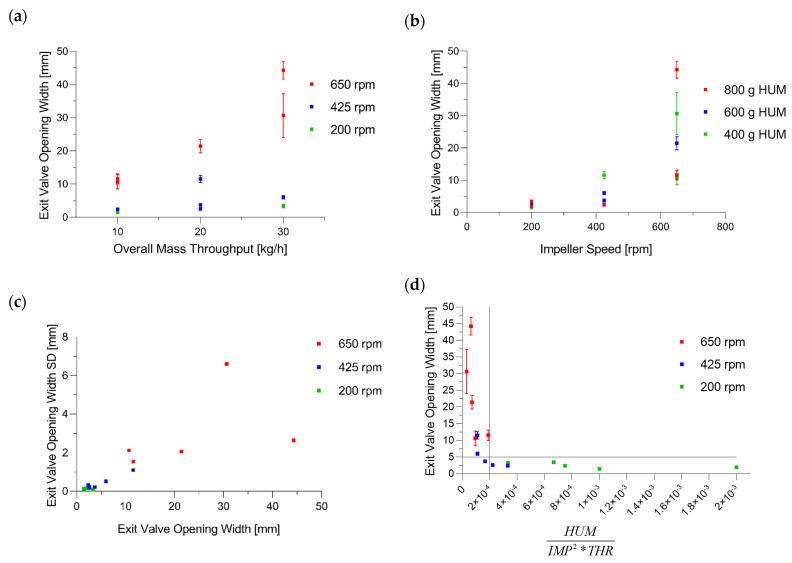
(**a**) Exit valve opening width vs. throughput (kg/h) in relation to varying impeller speeds. (**b**) EV in dependence of impeller speed. (**c**) EV SD vs. EV. (**d**) EV as function of $\frac{\mathrm{HUM}\;\left[g\right]}{{\mathrm{IMP}}^{2}\;[{\mathrm{rpm}}^{2}]*\mathrm{THR}\;\left[\frac{\mathrm{kg}}{h}\right]}$ where x-values higher than 2 × 10^−4^ result in EV below 5 mm.

In this regard, Figure 11a shows the exit valve opening width dependent on overall mass throughput, wherein increasing the throughput led to an increasing opening width. Furthermore, all EV at 650 rpm were higher than 10 mm. Figure 11b shows the exit valve depending on impeller speed. It confirms that the high impeller speed was an important reason for an increasing EV, while variations in HUM seemingly did not impact the exit valve (0.042 *p* = 0.874). Furthermore, a contour plot is used to demonstrate the significance of both model terms throughput and impeller speed (Figure 10). To determine suitable CMT settings based on this plot, small exit valve opening widths (<5 mm) are preferable, which is in line with the findings of Toson [8]. Additionally, data regarding blend potency SD confirmed the maximum of 5 mm opening value of the exit valve (further details below). 

**Figure 12:**
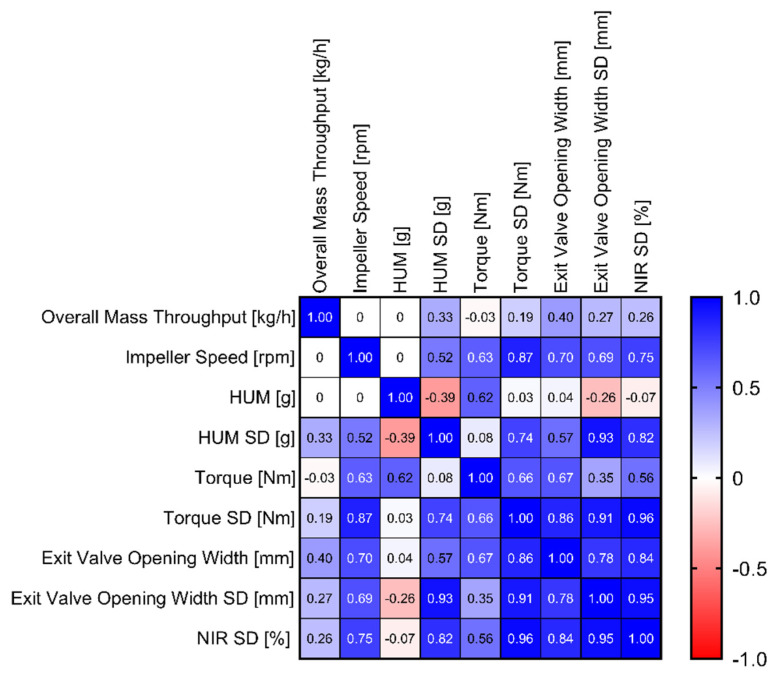
Correlation matrix of input variables and mixing parameters.

Regarding EV SD, Table 6 reveals that impeller speed was the only significant model term (Q^2^ = 0.822 R^2^ = 0.933). Furthermore, Figure 11c shows the EV standard deviation as a function of the EV opening width (0.785 *p* = 0.0002). This correlation leads to the conclusion that higher EV values increased the risk of a fluctuating opening width, impacting the variability of the blend potency values (0.952 *p* < 0.0001) and subsequently affecting content uniformity of the tablets. A correlation matrix with downstream parameters concerning the EV is shown in Figure 12.

Figure 11d shows a correlation of the EV with the ratio of $\frac{\mathrm{HUM}\;\left[g\right]}{{\mathrm{IMP}}^{2}\;[{\mathrm{rpm}}^{2}]\;*\mathrm{THR}\;\left[\frac{\mathrm{kg}}{h}\right]}$. This empirically found normalization revealed good processing for values exceeding 2 × 10^−4^.

As the decreased impeller speed proved to have the highest impact on reducing the exit valve opening width, it is certainly the primary parameter for reducing the EV value below 5 mm. However, one needs to consider the impact on the powder attributes of the blend, because a decrease in impeller speed will decrease the TBP and, therefore, the amount of lubrication. As described in the following sections, the TBP impacts the CBD, FRI, d_10_ values, FD and TS.

##### HUM SD

HUM is an essential variable in MRT and TBP (Equations (1) and (2)), and this is why it is crucial to choose suitable blender parameters to maintain a consistent process. Accordingly, the fluctuation in HUM led to variabilities in the MRT and TBP.

**Figure 13:**
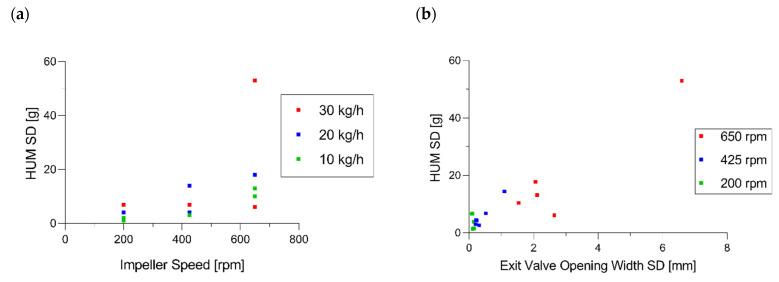
(**a**) HUM standard deviation as a function of impeller speed. (**b**) Dependencies between SD in EV and HUM.

Figure 13a shows the HUM SD as a function of impeller speed (0.514 *p* = 0.035). It reveals that the HUM standard deviations were not directly impacted by throughput. However, throughput is a significant model term, since comparatively low HUM standard deviations were obtained at low throughputs. On the other hand, higher impeller speeds tended to result in a larger span of HUM SD, and this could be caused by an unfavorable powder bed shape, due to higher centrifugal forces, as described by Toson et al. [8].

Figure 13b shows that the previously mentioned EV SD correlated with HUM SD (0.929 *p* < 0.001). That could be traced back to the PID control loop between HUM and EV, where EV is a function of HUM process values in order to maintain mass_in_ = mass_out_. Therefore, if variability could be observed in the HUM, then it occurred in EV, as well. To avoid those fluctuations, we can rely on the previous section, where impeller speed is the recommended parameter to control the corresponding process parameters. A detailed example is given in Supplementary Section E, “Additional Demonstration of HUM SD”. 

##### Torque of Lower Impeller

Regarding Table 6, the models for T_L_ (Q^2^ = 0.851 and R^2^ = 0.916) and T_L_ SD (Q^2^ = 0.882 and R^2^ = 0.949) could be considered good models. The low model validity for torque SD is, again, caused by low variability in the replicated center points, and, therefore, it is not a cause for concern. Basically, the torque represents the required energy to turn the impeller within the CMT and can be used to monitor the mixing process [43].

**Figure 14:**
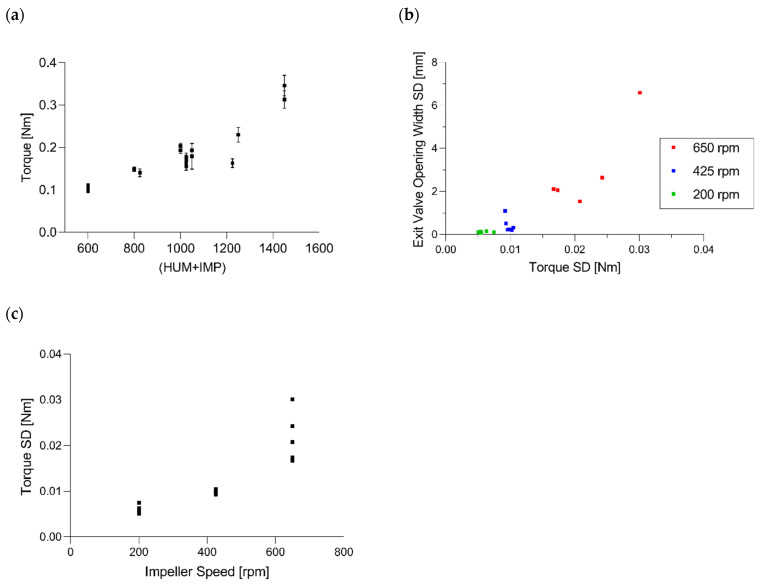
(**a**) Torque of the lower impeller as a function of the sum of HUM and IMP. (**b**) Correlation between variability in torque and exit valve opening width. (**c**) Impact of impeller speed on the torque values.

Since the model terms in Figure 5 showed similar coefficients of HUM and impeller speed, the torque could be seen as a function of the sum of both factors (0.888 *p* < 0.0001) (Figure 14a).

Figure 14b demonstrates the linearity between T_L_ SD and EV SD (0.906 *p* < 0.0001). The correlation between these standard deviations is based on the impact of impeller speed (IMP—T_L_ SD: 0.874 *p* < 0.0001), wherein the higher impeller rotation resulted in higher variabilities in both parameters (Figure 5 and Figure 14c).

Since standard deviations in both the torque and exit valve were strongly correlated, it is recommended to only focus on the EV values if monitoring is required. 

##### Blend Potency SD

Reflecting previously described process parameters, the correlations between blend potency SD and EV (0.843 *p* < 0.0001), EV SD (0.952 *p* < 0.0001), HUM SD (0.817 *p* < 0.0001), T_L_ SD (0.965 *p* < 0.0001) and IMP (0.753 *p* = 0.0005) could be observed (Figure 12). 

**Figure 15:**
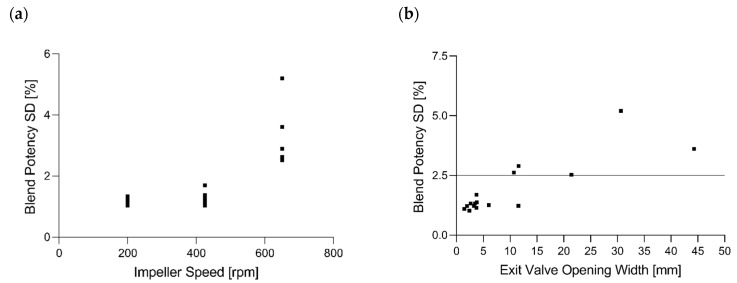
Blend Potency SD as a function of (**a**) impeller speed and (**b**) exit valve opening width.

Higher exit valve opening widths implicate that the powder bed was not entirely closed at the bottom of the CMT and that particles newly entering the CMT could exit unmixed [8]. Consequently, blend potency SDs and, therefore, blend inhomogeneities could be explained by insufficient mixing based on the structure of the powder bed within the blend. Figure 15a shows the blend potency standard deviation as a function of impeller speed, wherein all values at 650 rpm were above 2.5%. This observation could also be confirmed by using Figure 5, wherein IMP was the significant model term. Thus, to reduce blend potency SDs and, therefore, improve blend homogeneity and content uniformity of the tablets, reduction of the impeller speed is again proposed.

Figure 15b shows that all blend potency values obtained at exit valve opening widths below 10 mm were smaller than 2.5%. To minimize the risk of a higher blend potency SD, the presented results confirm maximum EV values below 5 mm.

Furthermore, independent of the blender variables, a potential risk for blend potency inhomogeneity could be adhesion of API at the walls due to electrostatic charging of particles [44]. 

#### 3.2.2. Material Attributes of the Blend

During continuous mixing with a vertical blender, TBP is the decisive factor in describing the impact on material attributes since magnesium stearate will be mixed simultaneously throughout the entire mixing process. It is the combination of impeller speed and blend time (MRT) (Equation (2)), governing shear and mixing intensity of the lubricant into the blend. For improvements regarding EV position and HUM uniformity following changes in material attributes must be considered:

Higher TBP represents more contact between impeller and powder particles and hence, it is implied that lubricant can be distributed more homogeneously into the blend with the potential risk of film formation. This would impact the tablet tensile strength and will be further discussed in section Tensile Strength.

##### Powder Density

With more impeller revolutions, more cavities of particles and granules can be filled and a layer around the particles can be built. On one hand, that increases the weight without increasing the volume and on the other hand, it is reducing particle-particle frictions due to the reduced friction of magnesium stearate filmed particles. Particles can now arrange more compactly, increasing the powder density [45,46]. 

**Figure 16:**
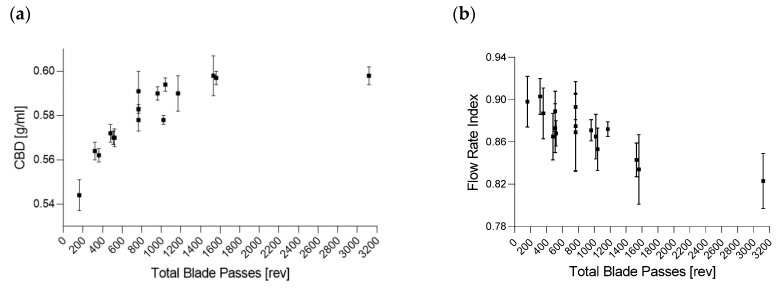

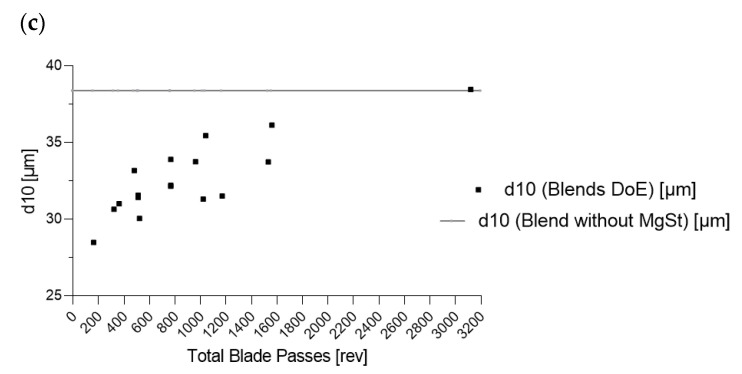
(**a**) Conditioned bulk density (CBD) (g/mL), (**b**) flow rate index (FRI) and (**c**) particle size (d_10_) (µm) as a function of total blade passes.

Figure 16a demonstrates an exponential relationship between TBP and CBD asymptotically reaching a value of 0.598 $\frac{g}{\mathrm{mL}}\;$ at 1560 TBP. At extreme values, such as 3120 revolutions, powder density will not increase any further and a maximum seemed to be reached, which led to the conclusion that increasing TBP only affected the material up to a certain limit.

Considering Figure 6 and the equation for TBP (Equation (2)), the significant model terms of the DoE revealed the same information, where higher values in HUM and impeller speed increased CBD, and higher throughputs decreased the powder density (Q^2^ = 0.735 and R^2^ = 0.850).

##### Flow Rate Index

Figure 16b shows the flow rate index (FRI) as a function of TBP (−0.846 *p* < 0.0001). In contrast to CBD, the FRI decreased with the rising TBP. Due to an increasing lubrication effect at the higher TBP, less energy was needed to move the blade through the powder bed, since the required energy is based on the resistance at the downward motion. Again, a plateau could be observed wherein the increasing TBP did not necessarily impact the FRI any further. The MLR analysis showed a model fit of Q^2^ = 0.800 and R^2^ = 0.896. 

##### Particle Size 

The description of density changes based on TBP also applies to the particle size (d_10_) (Figure 16c). At a high TBP, more magnesium stearate adhered to the particles, leading to a lower amount of the remaining free MgSt particles within the blend, and thus increasing the d_10_ values (0.836 *p* < 0.0001). As a reference, a blend without magnesium stearate was mixed by using a Turbula blender (Willy A. Bachofen AG, Muttenz, Switzerland), where a d_10_ value of 38.38 µm was obtained (Figure 16c). 

Therefore, the appearance of smaller particle sizes in the blend could be traced back to MgSt. As seen at 3120 revolutions, the d_10_ value was similar to the blend without MgSt, implicating that the fine fraction of MgSt was almost completely attached to the remaining raw materials at the higher TBP. Moreover, particle-size changes due to destruction of particles could be ruled out. In this case, the d_10_ values would have decreased with a higher shear.

Regarding the DoE results in Figure 6, a good model for d_10_ values could be obtained (R^2^ = 0.842 and Q^2^ = 0.587). Particle sizes of raw materials and blends are shown in Supplementary Table S34.

#### 3.2.3. Tableting Parameters

##### Fill Depth

**Figure 17:**
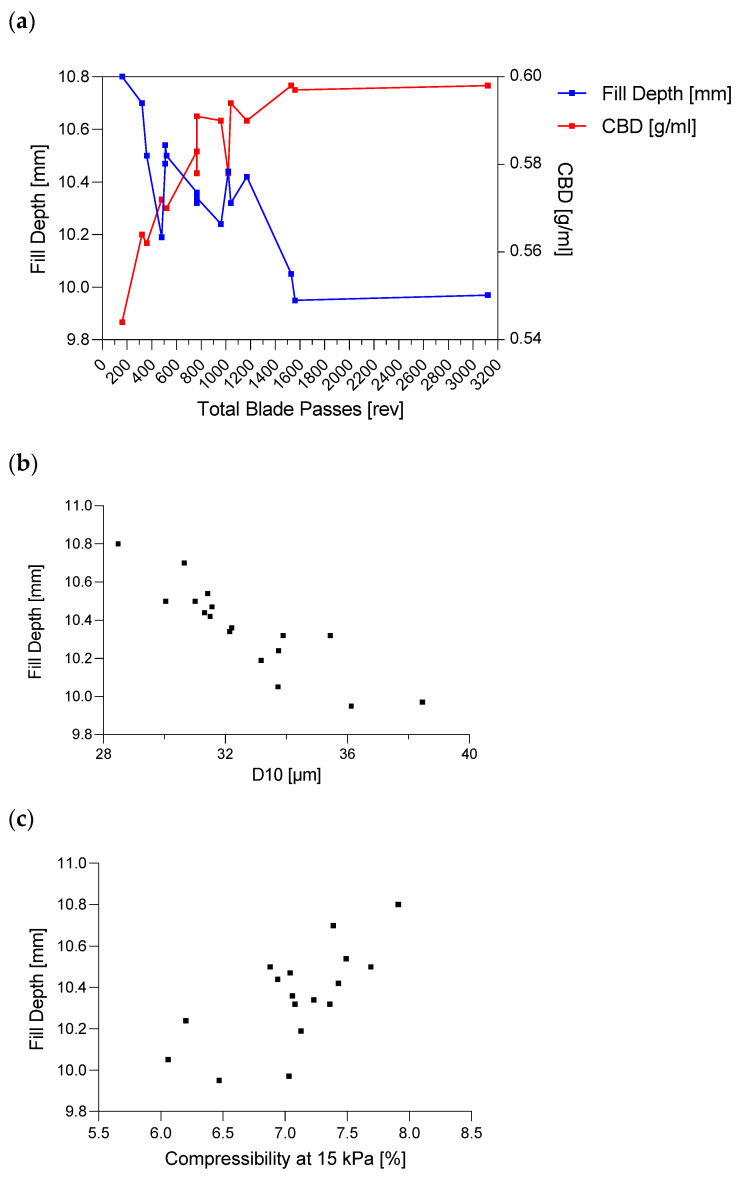
(**a**) Fill depth as function of total blade passes compared to bulk density. (**b**) Linearity between fill depth and d_10_ values. (**c**) Impact of compressibility on fill depth.

Higher powder density (CBD) will result in lower fill depths to fulfill the weight requirements, which could be confirmed in this paper (−0.844 *p* < 0.0001). As described above, the density of the blend was a function of TBP; that was why the fill depth was adjusted according to changes in TBP (−0.775 *p* < 0.0001), as well. Figure 17a shows the comparison between CBD and fill depth in dependence of TBP, where the increasing TBP resulted in higher density values and therefore in lower required fill depths. According to the TBP in Equation (2) and DoE Results in Figure 7, this observation could be confirmed since impeller speed was shown as negative and throughput as positive model term on fill-depth values. 

As already described, after a specific amount of revolutions, neither CBD nor FD values showed further changes with increasing TBP.

Figure 17b shows the fill depth as a function of particle size (d_10_). In general, smaller particle sizes are considered to decrease essential flowability, impacting a complete fill of the dies [47]. 

Regarding the die-filling process described by Xie and Puri [48], for powders with smaller particles, it is more challenging to lose entrained air due to cohesion during filling. Therefore, more volume and higher fill depths are required.. In this work, the correlation could be traced back again to lubrication, as described before, and not to cohesion (−0.224 *p* = 0.387).

Osorio and Muzzio [49] showed that higher powder compressibility values increase weight variability during capsule filling. Additionally, capsule weight decreased as powder compressibility increased. The same principle applies for die filling in this study, where higher powder compressibility led to higher fill depth values (0.703 *p* < 0.002) (Figure 17c). This observation may also be helpful if a capsule machine were used instead of a tablet press in continuous downstream processing.

##### Ejection Force

The ejection force is the required force to eject the tablet from the die and depends on the friction between the tablet and the die walls. Consequently, the reduction in ejection force is mainly influenced by the lubrication of the powder [50]. Usually, high ejection forces are accompanied by tableting problems and may cause damages to the tooling [51,52]. 

Regarding this dataset, the model-terms throughput and impeller speed shared the same deflections as for the fill depth (Figure 7); that means, it is indicated that a higher TBP results in a higher lubrication and lower ejection forces. However, although a strong correlation between ejection force and TBP was expected, only a correlation between ejection force and tablet-weight variability could be found (0.787 *p* = 0.0002). Nevertheless, a robust model regarding ejection force could be obtained by an MLR analysis (Q^2^ = 0.892 and R^2^ = 0.944). For further explanation regarding TBP and ejection force, see Supplementary Section G, “Ejection Force”.

#### 3.2.4. Tablet Properties

Even if good models for TS, TW and TT could be found, only few correlations regarding TW and TT could be obtained (see paragraph “Ejection Force”). 

##### Tensile Strength

**Figure 18:**
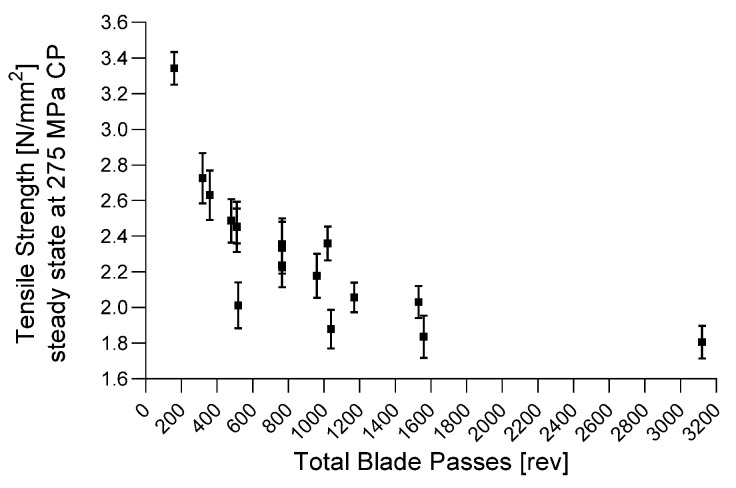
TS as a function of TBP at 275 MPa compression pressure.

Figure 18 demonstrates the tensile strength (TS) as a function of TBP (−0.704 *p* = 0.002), wherein a higher TBP resulted in lower tensile strength at the same compression pressure (275 MPa), due to increased lubrication efficiency. According to the DoE results in Figure 8, the significant model terms corresponded to the TBP Equation (2), where a higher throughput, lower HUM and lower impeller speed result in a lower TBP and, therefore, in higher tensile strengths of the tablet. If previous process states need to be optimized by adapting CMT parameters, a similar TBP should be maintained to ensure the correct TS. 

Again, after 1560 revolutions, a plateau was reached, and no further reduction in tensile strength could be noticed with the increasing TBP.

##### Compression-Force Profile

A compression-force profile was conducted by using 118, 157, 169, 236 and 275 MPa compression pressure. During phase 16, no compression-force profile could be performed, because HUM increased from 0.8 to ~1.1 kg and the exit valve opened up to 45 mm, without any chance of decreasing. Thus, a consistent process flow could not be reached, and the correct setting of the FD and compression pressure was not possible.

**Figure 19:**
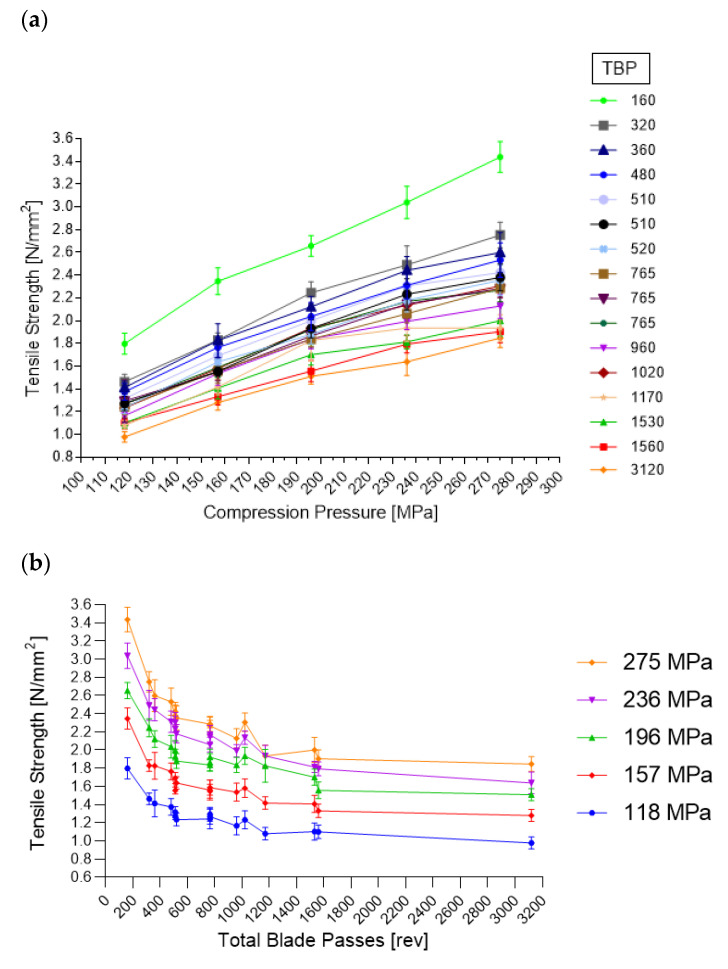
(**a**) Overview of all phases (TBP) regarding compression pressure and tensile strength. (**b**) Overview of all compression pressures and the corresponding tensile strength based on the lubrication (TBP).

Figure 19 includes the TS as a function of the corresponding compression pressure and TBP. Figure 19a demonstrates the profiles of each phase as a function of compression pressure, wherein the lowest TBP showed the highest values. Figure 19b reflects the TS as a function of TBP, where higher compression pressure led to profiles with higher values. Table 10 shows the fit statistics regarding tensile strengths obtained during the compression-force profiles.

## 4. Sweet Spot

**Figure 20:**
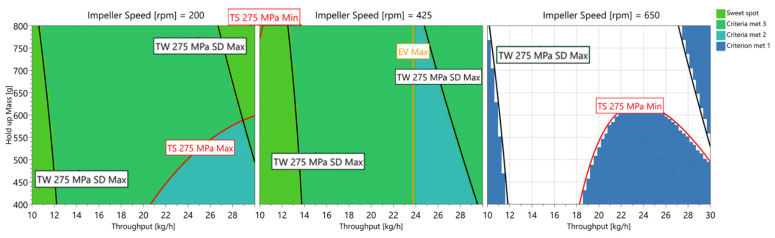
Sweet spot (light green) reveals the combination of the DoE input variables in which the criteria are met. The color of the borders indicate which criterion is not met anymore. Black borders = TW SD, red borders = TS and orange boarders = EV.

By using MODDE, it is possible to detect a sweet spot where several criteria are met. For this paper, exit valve opening width (1–5 mm), blend potency SD (0–3%), tensile strength (2–3 MPa) and tablet-weight variability (0–2.5 mg) are considered critical parameters. In brackets, the favorable process values are shown. Figure 20 shows a visualization of a combination of input variables (throughput, hold-up mass and impeller speed) in which all criteria are met (light green). At an impeller speed of 650 rpm, no sweet spot could be achieved. With reducing impeller speeds, sweet spots at low throughputs are possible at 425 and 200 rpm. At 200 rpm, sweet spots could be achieved at low throughputs independently of HUM. Since it is preferred to run the process with higher throughputs, an optimal setting for this formulation can be observed at a combination of high throughputs and high HUM values at 200 rpm impeller speed. 

## 5. Conclusions

This paper showed the evaluation of the downstream process states based on throughput, hold-up mass and impeller speed in a continuous direct compression line, including a single blending step, in a vertical blender (CMT). For all settings in the performed DoE, the same composition and compounds were used, so that the initial material attributes and lubrication sensitivity remained constant. 

In this study, the model terms of the process states based on the CMT parameters were evaluated by means of a MLR analysis. Corresponding fit statistics are shown in Table 11.

Furthermore, the connections between the parameters were evaluated. Regarding mixing parameters, it has been shown that the exit valve opening width and variability in exit valve, in hold-up mass, in torque and in blend potency are significantly correlated and can all be controlled mainly by impeller speed. If the improvement of these parameters is required, it needs to be considered that changes in impeller speed will also lead to changes in TBP.

With higher TBP, more shear is transmitted to the powder and more magnesium stearate will adhere to the remaining particles, leading to more lubrication and higher variation in material attributes. Hence, TBP significantly correlated with the blend’s material attributes (density, d_10_ values and flow-rate index), the fill depth and the tensile strength of the tablets. 

Target criteria (exit valve opening width (1–5 mm), blend potency SD (0–3%), tensile strength (2–3 MPa) and tablet-weight variability (0–2.5 mg)) could generally be found at impeller speeds between 200 and 425 rpm and at throughputs between 10 and 12 kg/h independent of HUM. To run the process as fast as possible, high throughput, high HUM and 200 rpm IMP are required to fulfill the target criteria and, therefore, represent the optimal setting for this formulation.

## Figures and Tables

**Table 1:** Overview of considered responses, where HUM, IMP and THR were adjusted as input variables.

Responses	
Mixing parameters	T_L,_
	EV
	Blend potency as predicted by the NIR model
Material attributes of the blend	FRI
	Particle size (d_10_)
	CBD
Tableting parameters	FD
	BCH
	EF
Tablet properties	TS
	TT
	TW

**Table 2:** DoE settings, where phase 7, 9 and 11 are the replicates of the center point.

Phase	Throughput (kg/h)	Hold-Up Mass (g)	Impeller Speed (rpm)	MRT (min)	TBP (rev)
1	10	400	200	2.4	480
2	10	400	650	2.4	1560
3	10	600	425	3.6	1530
4	10	800	200	4.8	960
5	10	800	650	4.8	3120
6	20	400	425	1.2	510
7	20	600	425	1.8	765
8	20	600	200	1.8	360
9	20	600	425	1.8	765
10	20	800	425	2.4	1020
11	20	600	425	1.8	765
12	20	600	650	1.8	1170
13	30	400	200	0.8	160
14	30	400	650	0.8	520
15	30	600	425	1.2	510
16	30	800	650	1.6	1040
17	30	800	200	1.6	320

**Table 3:** Feeder settings for each raw material.

	Microcrystalline Cellulose	Saccharin Sodium Monohydrate	Calcium Di-Phosphate	Sodium Starch Glycolate	Magnesium Stearate
Composition (%)	49.104	21.844	24.552	3	1.5
Top-Up Volume (L)	1.6	1.2	1.6	1.2	0.8
Gearbox Type	1 (63:1)	2 (235:1)	2 (235:1)	3 (455:1)	3 (455:1)
Screw Pitch (mm)	20	10	20	10	20
Refill Level (dm^3^)	0.5	0.74	0.3	0.25	1.5

**Table 4:** Classification of Carr Index [30].

Flowability	Carr’s Index
Excellent	<15
Correct	15–25
Poor	>25

**Table 5:** Interpretation of Pearson correlation coefficients [42].

Correlation Coefficient	Interpretation
0.9 to 1.0 (−0.9 to −1.0)	Very high correlation
0.7 to 0.9 (−0.7 to −0.9)	High correlation
0.5 to 0.7 (−0.5 to −0.7)	Moderate correlation
0.3 to 0.5 (−0.3 to −0.5)	Low correlation
0.0 to 0.3 (−0.0 to −0.3)	Negligible correlation

**Table 6:** Overview of fit statistics regarding mixing parameters after removing non-significant model terms.

Response Factor	Data Transformation	Q^2^	R^2^	Adjusted R^2^
Exit Valve Opening Width	Logarithmic	0.860	0.905	0.883
Exit Valve Opening Width SD	Logarithmic	0.822	0.933	0.893
Torque Lower Impeller	Logarithmic	0.851	0.916	0.896
Torque Lower Impeller SD	Logarithmic	0.882	0.949	0.933
Blend Potency SD	Logarithmic	0.491	0.669	0.622
HUM SD	Logarithmic	0.428	0.727	0.664

**Table 7:** Overview of fit statistics regarding material attributes of the blend.

Response Factor	Data Transformation	Q^2^	R^2^	Adjusted R^2^
Conditioned Bulk Density	-	0.735	0.850	0.816
Flow Rate Index	-	0.800	0.896	0.848
Particle Size (d_10_)	-	0.587	0.842	0.747

**Table 8:** Overview of fit statistics regarding tablet-press parameters.

Response Factor	Data Transformation	Q^2^	R^2^	Adjusted R^2^
Fill Depth	-	0.873	0.941	0.914
Bottom Main Compression Height	-	0.774	0.928	0.885
Ejection Force	-	0.892	0.944	0.931

**Table 9:** Overview of fit statistics regarding tablet properties.

Response Factor	Data Transformation	Q^2^	R^2^	Adjusted R^2^
Tensile Strength	Logarithmic	0.907	0.976	0.958
Tensile Strength SD	-	−0.090	0.283	0.117
Tablet Weight	Logarithmic	0.641	0.904	0.847
Tablet Weight SD	-	0.472	0.856	0.770
Tablet Thickness	Logarithmic	0.718	0.953	0.917
Tablet Thickness SD	-	0.395	0.694	0.592

**Table 10:** Overview of fit statistics regarding tensile strengths obtained during compression-force profiles.

Response Factor	Data Transformation	Q^2^	R^2^	Adjusted R^2^
Tensile Strength at 118 MPa	Logarithmic	0.905	0.958	0.942
Tensile Strength at 157 MPa	Logarithmic	0.877	0.963	0.944
Tensile Strength at 169 MPa	Logarithmic	0.870	0.940	0.918
Tensile Strength at 236 MPa	Logarithmic	0.923	0.978	0.964
Tensile Strength at 275 MPa	Logarithmic	0.927	0.975	0.963

**Table 11:** Overview of the models obtained in this study.

Responses	Q^2^	R^2^	Adjusted R^2^
Exit valve opening width	0.860	0.905	0.883
Exit valve opening width SD	0.822	0.933	0.893
Torque of lower impeller	0.851	0.916	0.896
Torque of lower impeller SD	0.882	0.949	0.933
Conditioned bulk density	0.735	0.850	0.816
Flow rate index	0.800	0.896	0.848
Fill depth	0.873	0.941	0.914
Bottom main compression height	0.774	0.928	0.885
Ejection force	0.892	0.944	0.931
Tablet thickness	0.718	0.953	0.917
Tablet weight	0.642	0.904	0.847
Tensile strength	0.907	0.976	0.958

## Supplementary Materials

The following supporting information can be downloaded at https://www.mdpi.com/article/10.3390/pharmaceutics14020278/s1.

## Abbreviations

API	active pharmaceutical ingredient
BCH	bottom main compression height
BFE	basic flow energy
CBD	conditioned bulk density
CMT	continuous mixing technology
CP	compression pressure
DC	direct compression
DoE	design of experiment
EF	ejection force
EV	exit valve opening width
EV SD	exit valve opening width standard deviation
FD	fill depth
FRI	flow rate index
HUM	hold-up mass
IMP	impeller speed
LiW	loss in weight
MBM	mass balance model
MCC	microcrystalline cellulose
MgSt	magnesium stearate
MLR	multiple linear regression
MRT	mean residence time
NIR	near infrared
PCMM	portable, continuous, modular, miniature
PID	proportional–integral–derivative
PLS	partial least square
PV	process value
RTD	residence-time distribution
SE	specific energy
SI	stability index
SNV	standard normal variate
SD	standard deviation
TBP	total blade passes
THR	throughput
T_L_	torque lower impeller
T_L_ SD	torque lower impeller standard deviation
TS	tensile strength
TT	tablet thickness
TW	tablet weight
