# Supplementary material for: Impact of Vertical Blender Unit Parameters on Subsequent Process Parameters and Tablet Properties in a Continuous Direct Compression Line
Source: Pharmaceutics. 2022 Jan 25;14(2):278. doi: 10.3390/pharmaceutics14020278 (PMC8879867; doi:10.3390/pharmaceutics14020278)
Supplement: Supplementary file 1 [file pharmaceutics-14-00278-s001.zip › pharmaceutics-1525010-supplementary.pdf]

pharmaceutics

A. Summary of Fit: Mixing Parameters

P-Values <0.05 are considered as significant.

Table S1. Shows the experiment numbers and corresponding DoE Setting.

Experiment Number	throughput [kg/h]	hold up mass [g]	impeller speed [rpm]
1	10	400	200
2	30	400	200
3	10	800	200
4	30	800	200
5	10	400	650
6	30	400	650
7	10	800	650
8	30	800	650
9	10	600	425
10	30	600	425
11	20	400	425
12	20	800	425
13	20	600	200
14	20	600	650
15	20	600	425
16	20	600	425
17	20	600	425

A.1 Summary of Fit: Exit Valve Opening Width

Data Transformation: logarithmic

Low Model validity due to minimal variability in exit valve opening widths at the three replicates (3.706 -3.786 mm).

Model Equation:

$$Log_{10}(EV) = 0.125741 + 0.020304 * THR - 0.00164767 * IMP + 4.37955 * 10^{-6} * IMP^2$$

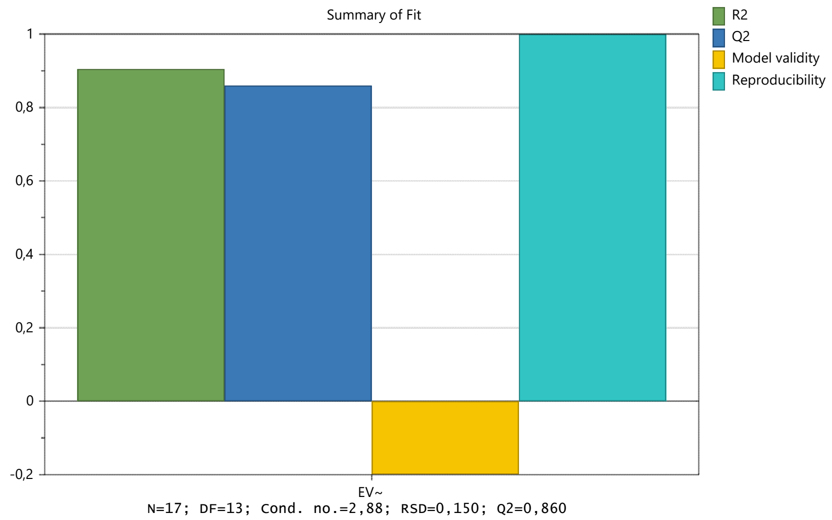

Figure S1. Summary of fit including R^2, Q^2, Model validity and Reproducibility.

Table S2. Fit statistics regarding Q^2, R^2 and R^2 adjusted.

Fit Statistics	
Q^2	0.860
R^2	0.905
R^2 adjusted	0.883

Table S3. Significance of the obtained model.

	Model	Lack of Fit
F-Value	41.2702	1170.91
P-Value	0.000	0.001
Significant?	yes	yes

<u>Residuals Normal Probability</u>

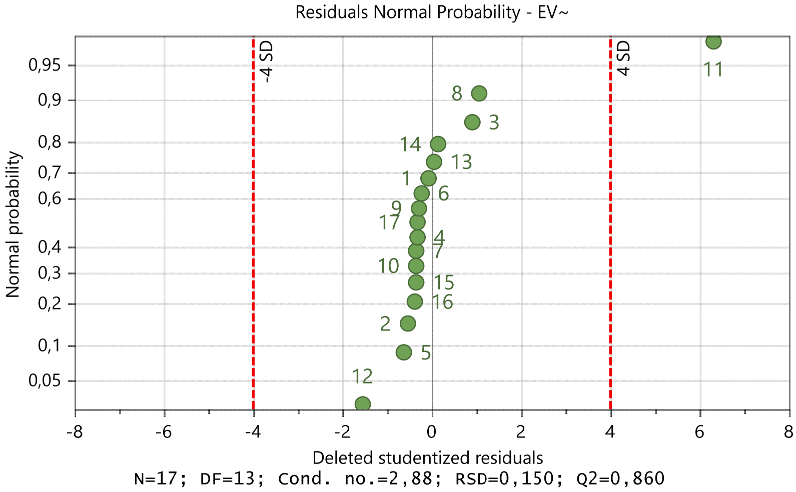

Figure S2. Residuals Normal Probability plot.

<u>Observed vs Predicted</u>

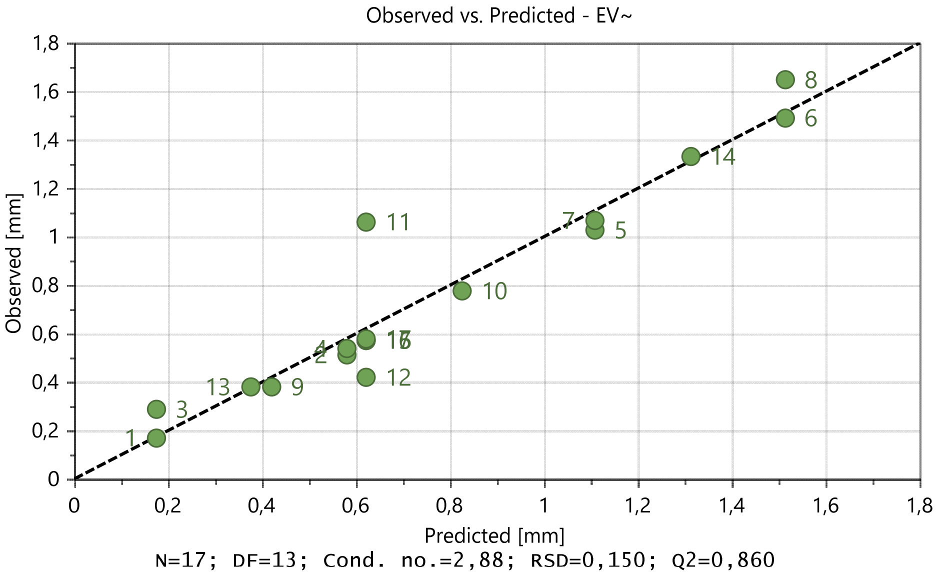

Figure S3. Comparison between observed and predicted results.

A.2 Summary of Fit: Exit Valve Opening Width StDev

Data Transformation: logarithmic

Low Model validity due to minimal variability in EV StDev at the three replicates (0.215 - 0.245 mm).

Model Equation:

$$Log_{10}(EVStDev) = -0.895478 - 0.0180823 * THR + 0.000444799 * HUM - 0.000454596 * IMP + 4.30254 * 10^{-6} * IMP^2 + 5.75986 * 10^{-5} * THR * IMP - 2.22512 * 10^{-6}\ HUM * IMP$$

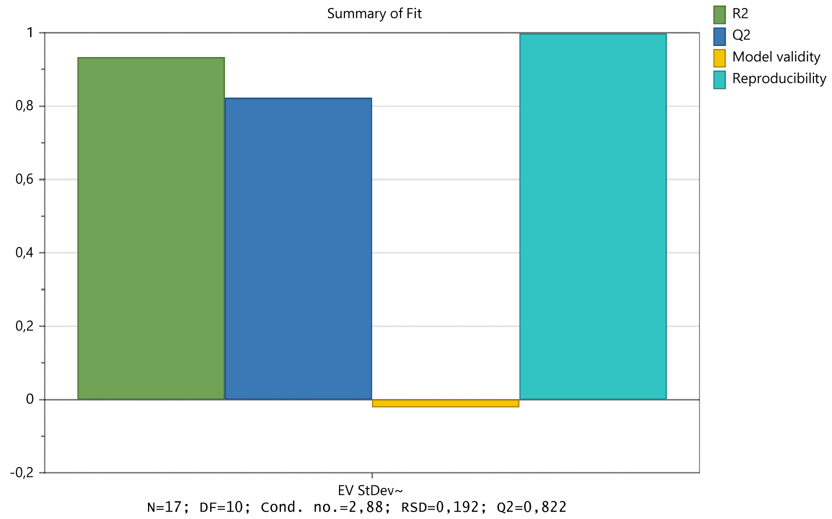

Figure S4. Summary of fit including R^2, Q^2, Model validity and Reproducibility.

Table S4. Fit statistics regarding Q^2, R^2 and R^2 adjusted.

Fit Statistics	
Q^2	0.822
R^2	0.933
R^2 adjusted	0.893

Table S5. Significance of the obtained model.

	Model	Lack of Fit
F-Value	23.2618	58.4081
P-Value	0.000	0.017
Significant?	yes	yes

Residuals Normal Probability

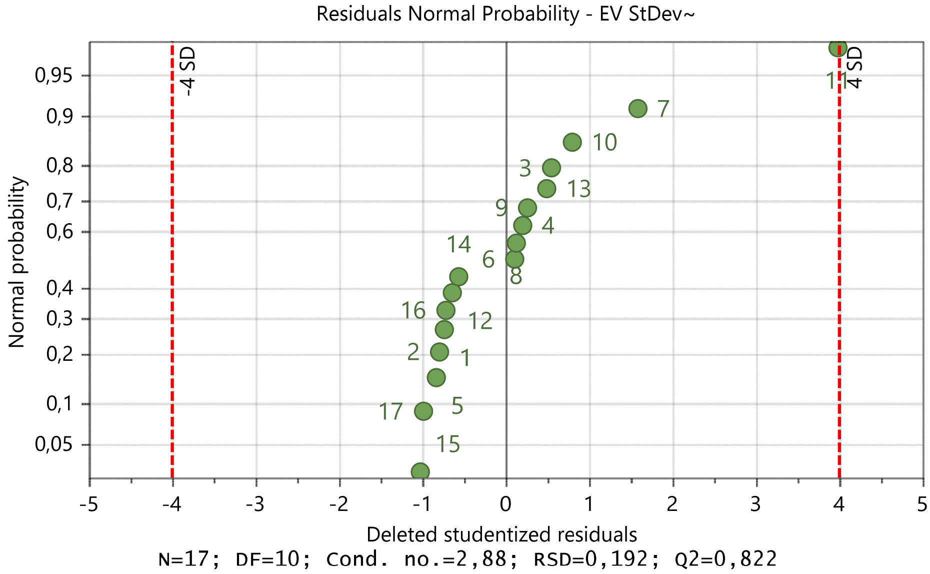

Figure S5. Residuals Normal Probability plot.

Observed vs Predicted

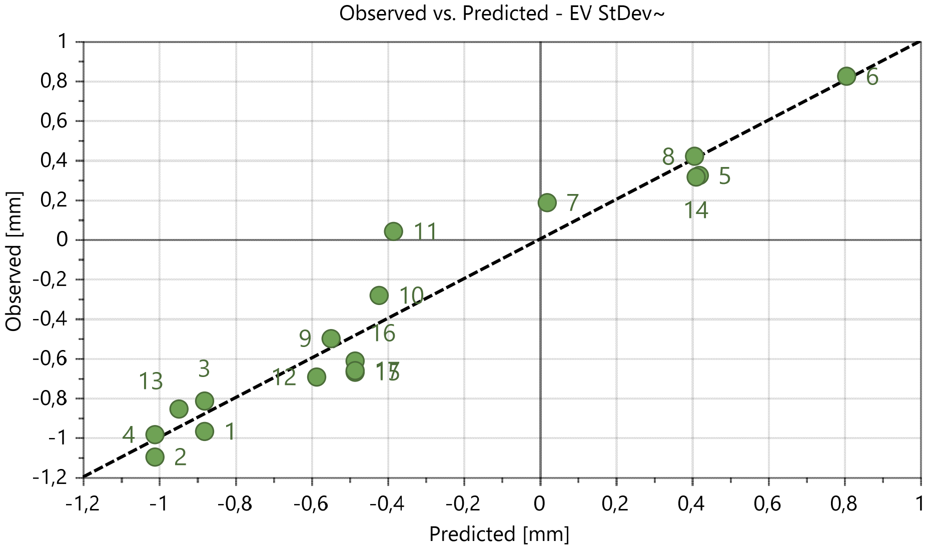

N=17; DF=10; Cond. no.=2,88; RSD=0,192; Q2=0,822

Figure S6. Comparison between observed and predicted results.

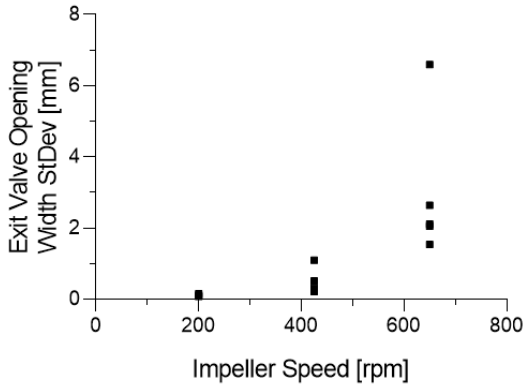

Figure S7. EV as function of impeller speed. Data transformation was conducted since variability is not constant.

A.3 Summary of Fit: Torque Lower Impeller

Data Transformation: logarithmic

Model Equation:

$$Log_{10}(T_L) = -1.11007 + 0.000560652 * HUM - 0.000591002 * IMP + 1.28941 * 10^{-6} * IMP^2$$

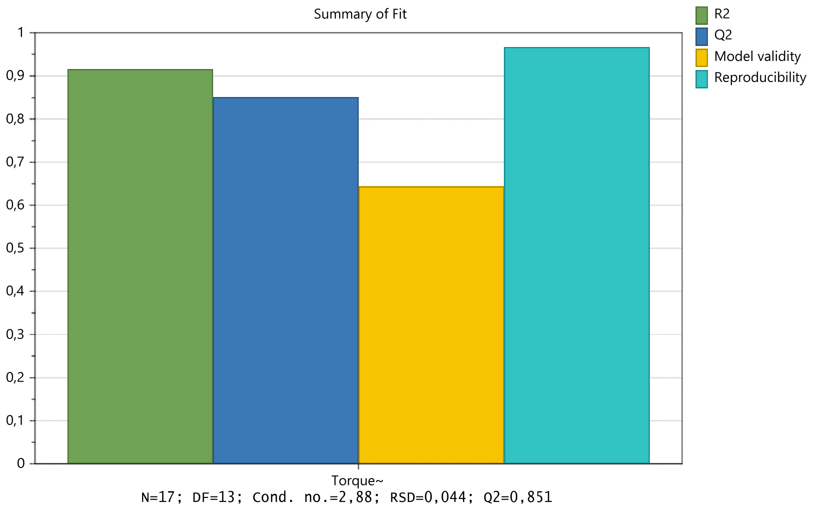

Figure S8. Summary of fit including R^2, Q^2, Model validity and Reproducibility.

Table S6. Fit statistics regarding Q^2, R^2 and R^2 adjusted.

Fit Statistics	
Q^2	0.851
R^2	0.916
R^2 adjusted	0.896

Table S7. Significance of the obtained model.

	Model	Lack of Fit
F-Value	47.0715	3.53695
P-Value	0.000	0.241
Significant?	yes	no

Residuals Normal Probability

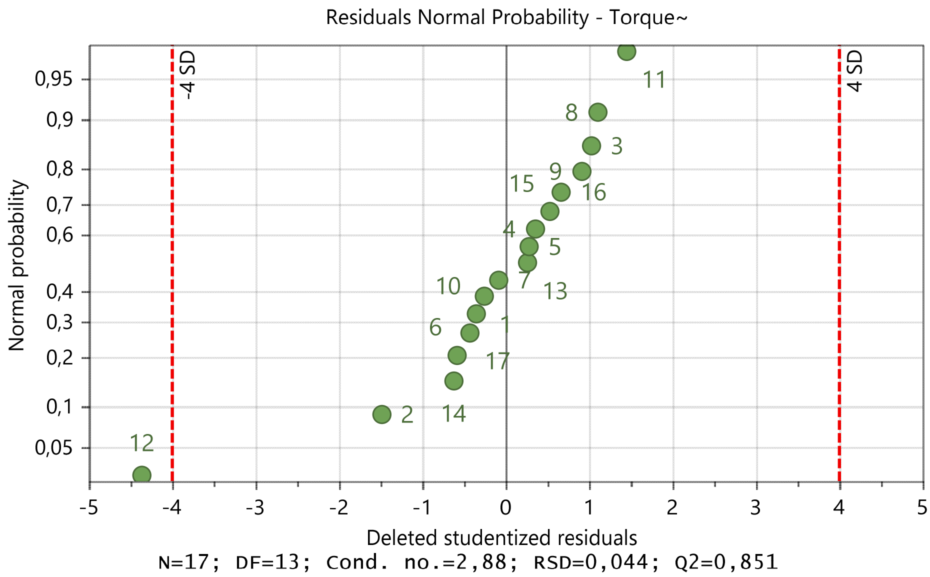

Figure S9. Residuals Normal Probability plot.

Observed vs Predicted

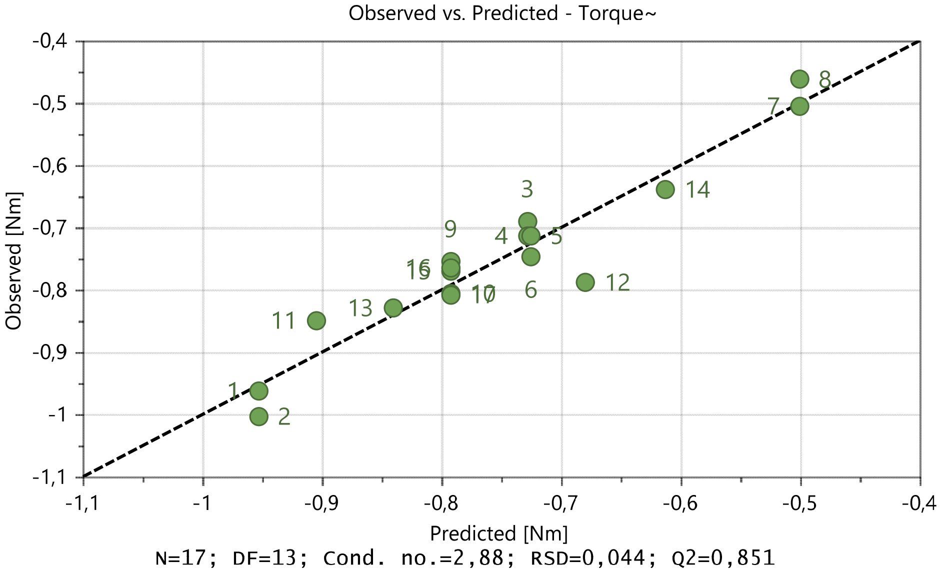

Figure S10. Comparison between observed and predicted results.

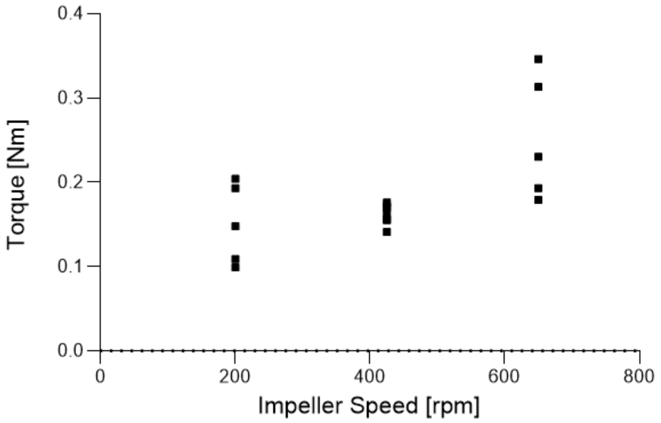

Figure S11. Torque of the lower impeller as function of impeller speed. Data transformation was conducted since variability is not constant.

A.4 Summary of Fit: Torque Lower Impeller StDev

Data Transformation: logarithmic

Low Model validity due to minimal variability in Torque StDev at the three replicates (0.0096-0.01 Nm).

Model Equation:

$$Log_{10}(T_L\ StDev) = -2.15687 + 0.00377409 * THR - 0.00165284 * HUM + 0.00123844 * IMP + 1.4924 * 10^{-6} * HUM^2$$

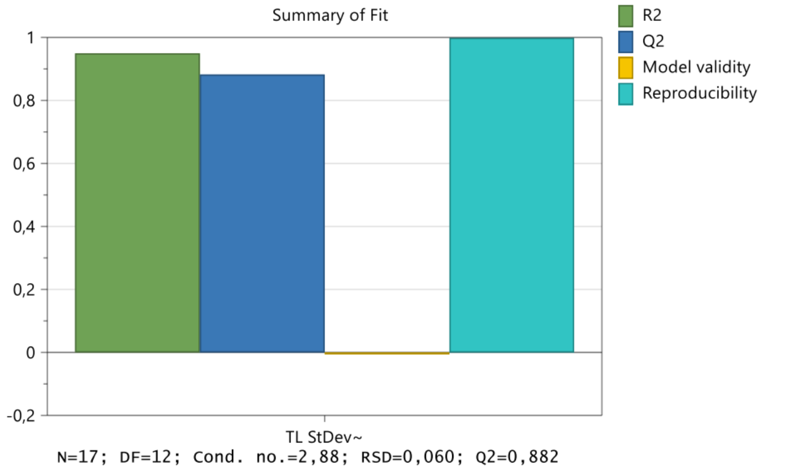

Figure S12. Summary of fit including R^2, Q^2, Model validity and Reproducibility.

Table S8. Fit statistics regarding Q^2, R^2 and R^2 adjusted.

Fit Statistics	
Q^2	0.882
R^2	0.949
R^2 adjusted	0.933

Table S9. Significance of the obtained model.

	Model	Lack of Fit
F-Value	56.2754	54.9535
P-Value	0.000	0.018
Significant?	yes	yes

<u>Residuals Normal Probability</u>

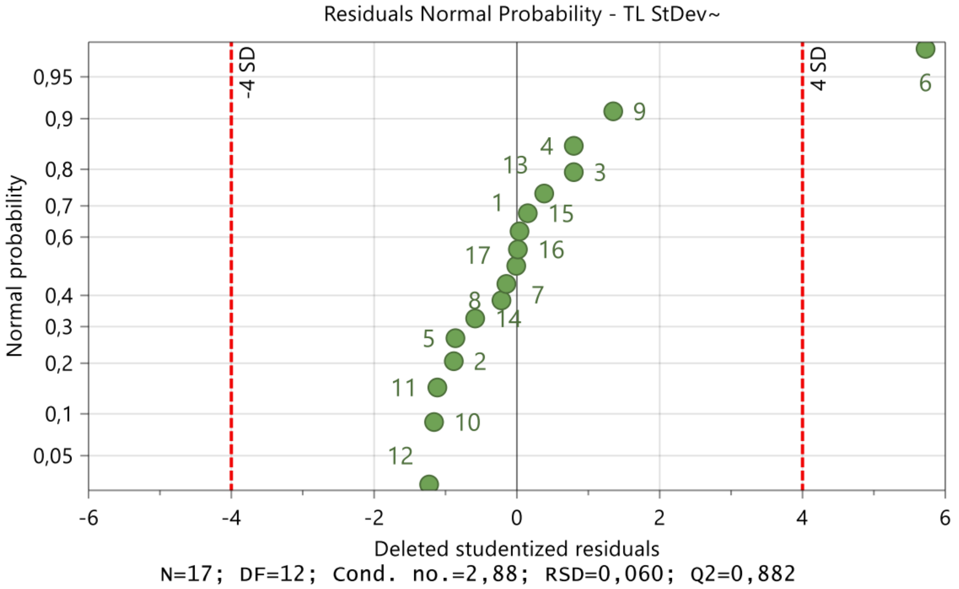

Figure S13. Residuals Normal Probability plot.

Observed vs Predicted

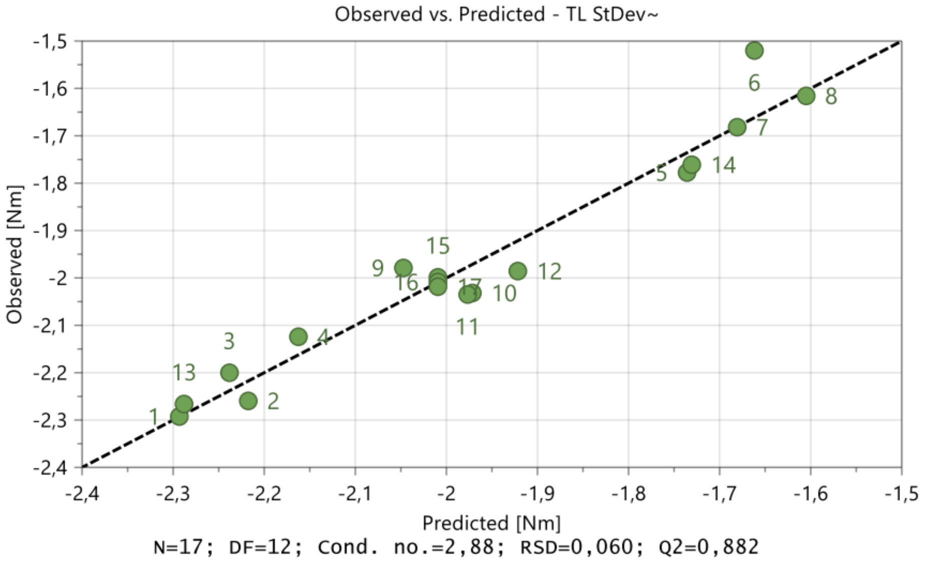

Figure S14. Comparison between observed and predicted results.

A.5 Summary of Fit: HUM StDev

Data Transformation: logarithmic

Low Model validity due to very small variability in HUM StDev at the three replicates (0.00426 – 0.00439 kg).

Model Equation:

$$Log_{10}(HUM\ StDev) = -4.62943 + 0.0875789 * THR + 0.00230934 * HUM + 0.00202194 * IMP + 3.31232 * 10^{-6} * IMP^2 - 7.08257 * 10^{-5}\, THR * HUM - 5.55935 * 10^{-5}\, THR * IMP - 3.78083 * 10^{-6}\, HUM * IMP$$

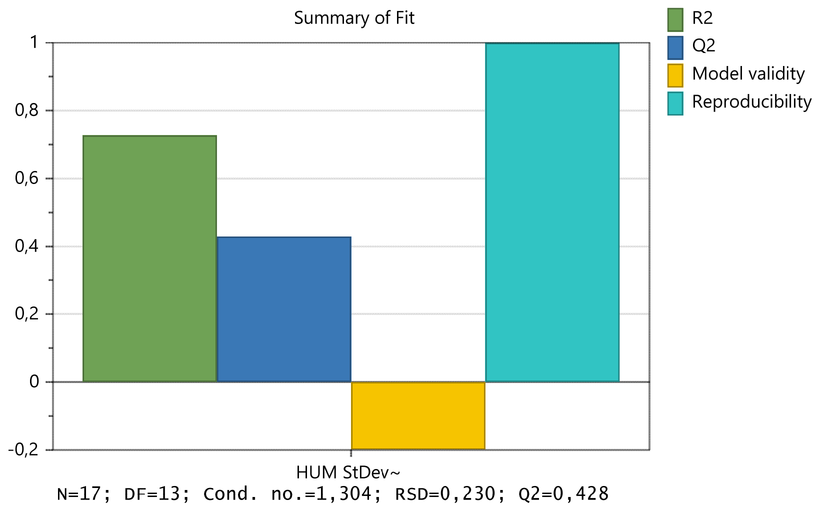

Figure S15. Summary of fit including R^2, Q^2, Model validity and Reproducibility.

Table S10. Fit statistics regarding Q^2, R^2 and R^2 adjusted.

Fit Statistics	
Q^2	0.428
R^2	0.727
R^2 adjusted	0.664

Table S11. Significance of the obtained model.

	Model	Lack of Fit
F-Value	11.5323	788.846
P-Value	0.001	0.001
Significant?	yes	yes

Residuals Normal Probability

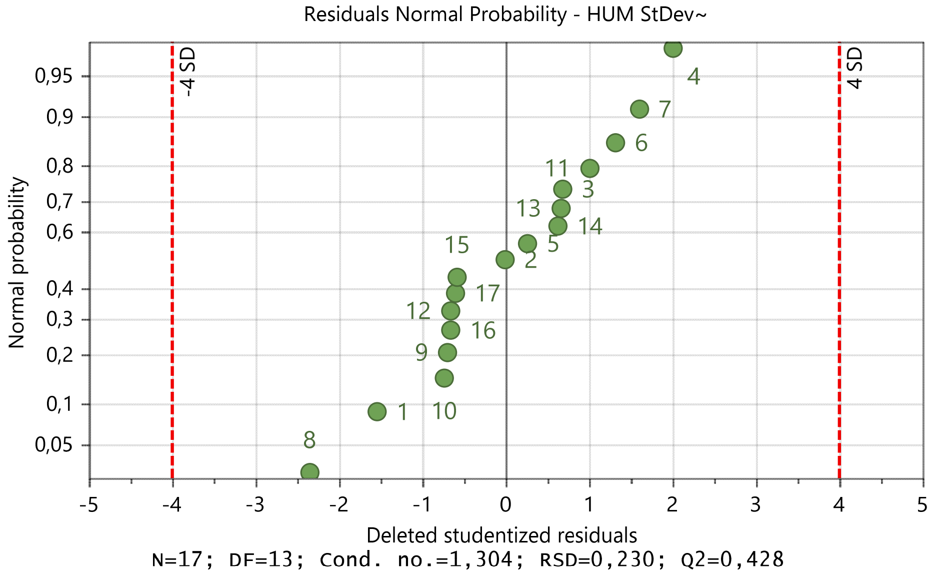

Figure S16. Residuals Normal Probability plot.

Observed vs Predicted

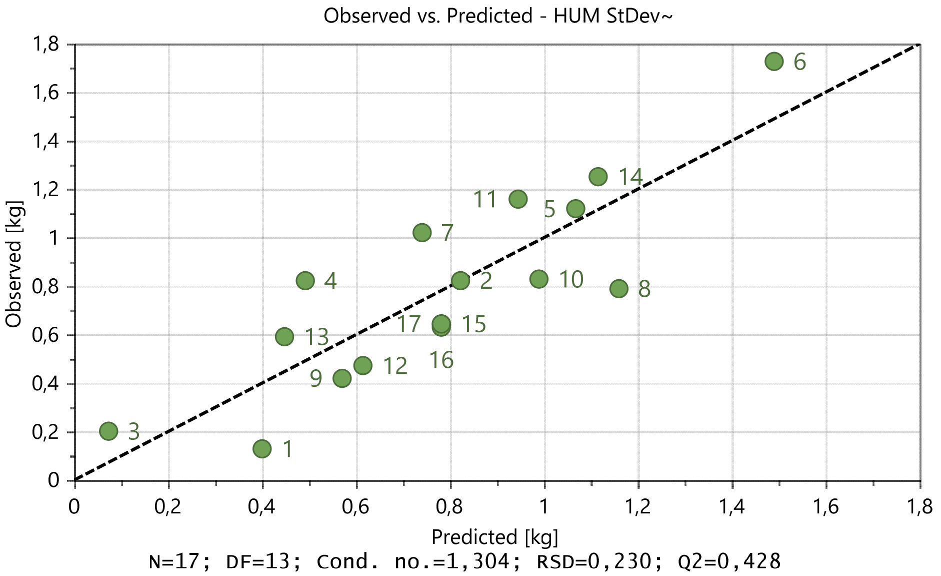

Figure S17. Comparison between observed and predicted results.

A.6 Summary of Fit: Blend Potency StDev

Data Transformation: logarithmic

Model Equation:

$$Log_{10}(Blend\ Potency\ StDev) = 0.655084 - 0.00330149 * IMP + 4.73778 * 10^{-6} * IMP^2$$

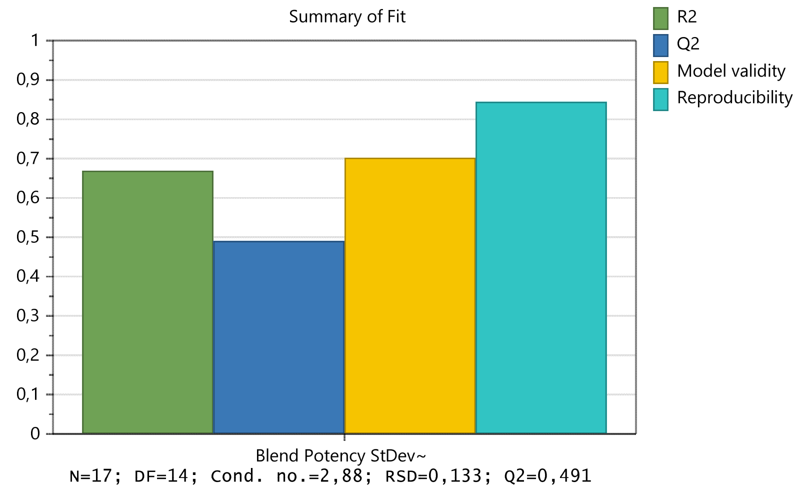

Figure S18. Summary of fit including R^2, Q^2, Model validity and Reproducibility.

Table S12. Fit statistics regarding Q^2, R^2 and R^2 adjusted.

Fit Statistics	
Q^2	0.491
R^2	0.669
R^2 adjusted	0.622

Table S13. Significance of the obtained model.

	Model	Lack of Fit
F-Value	14.1639	2.67007
P-Value	0.000	0.305
Significant?	yes	no

Residuals Normal Probability

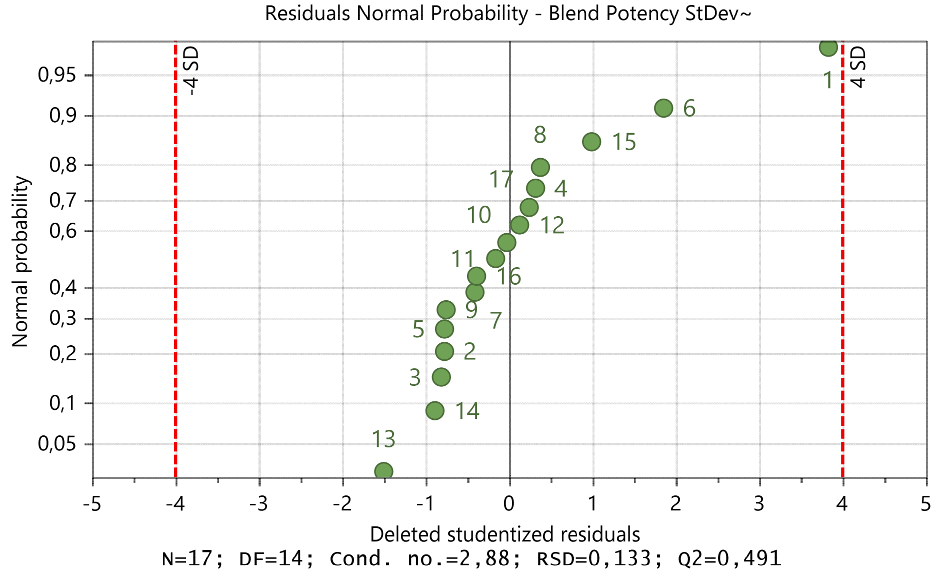

Figure S19. Residuals Normal Probability plot.

Observed vs Predicted

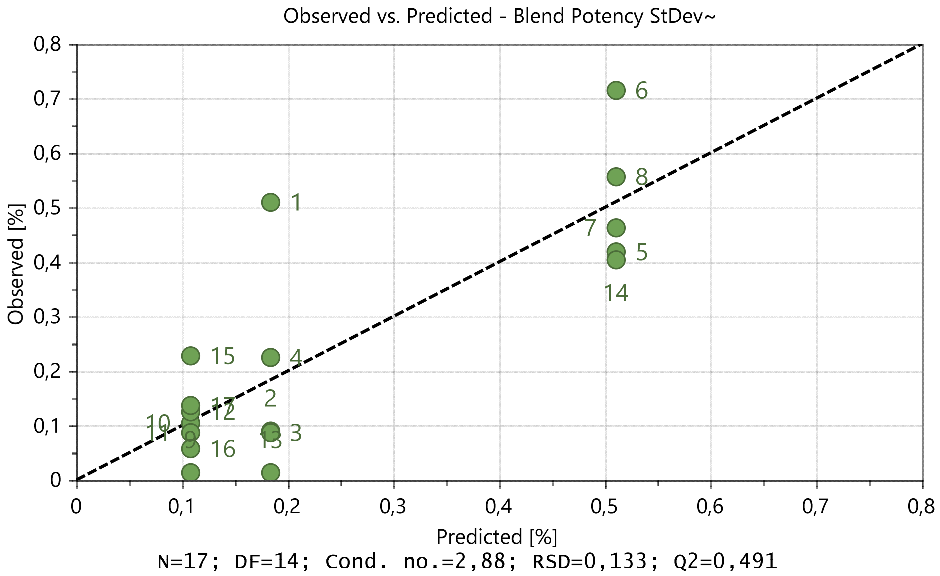

Figure S20. Comparison between observed and predicted results.

B. Summary of Fit: Material Attributes of the Blend

B.1 Summary of Fit: CBD

Data Transformation: -

Model Equation:

$$CBD = 0.558553 - 0.00113 * THR + 3.54999 * 10^{-5} * HUM + 5.2 * 10^{-5} * IMP$$

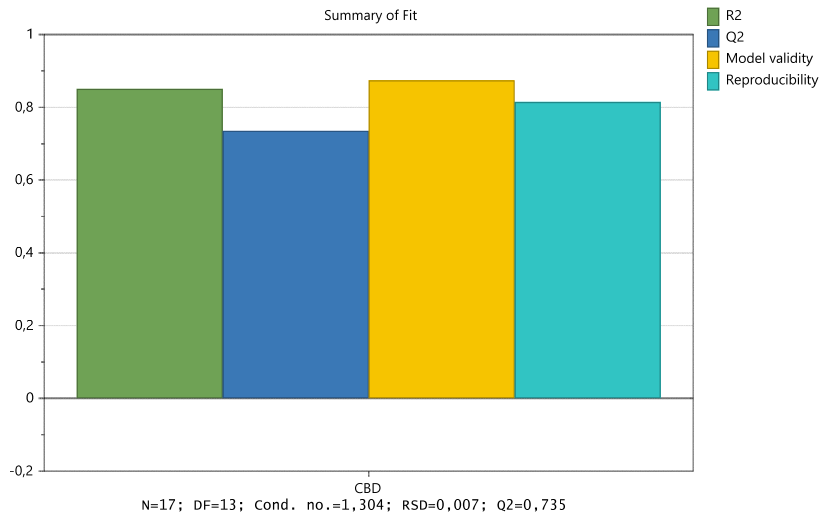

Figure S21. Summary of fit including R^2, Q^2, Model validity and Reproducibility.

Table S14. Fit statistics regarding Q^2, R^2 and R^2 adjusted.

Fit Statistics	
Q^2	0.735
R^2	0.850
R^2 adjusted	0.816

Table S15. Significance of the obtained model.

	Model	Lack of Fit
F-Value	24.639	0.98939
P-Value	0.000	0.605
Significant?	yes	no

Residuals Normal Probability

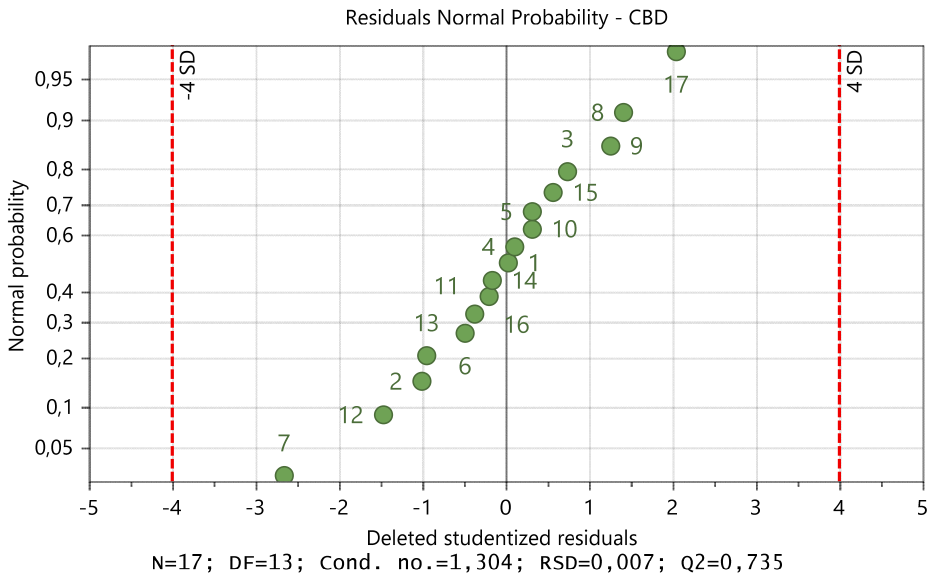

Figure S22. Residuals Normal Probability plot.

Observed vs Predicted

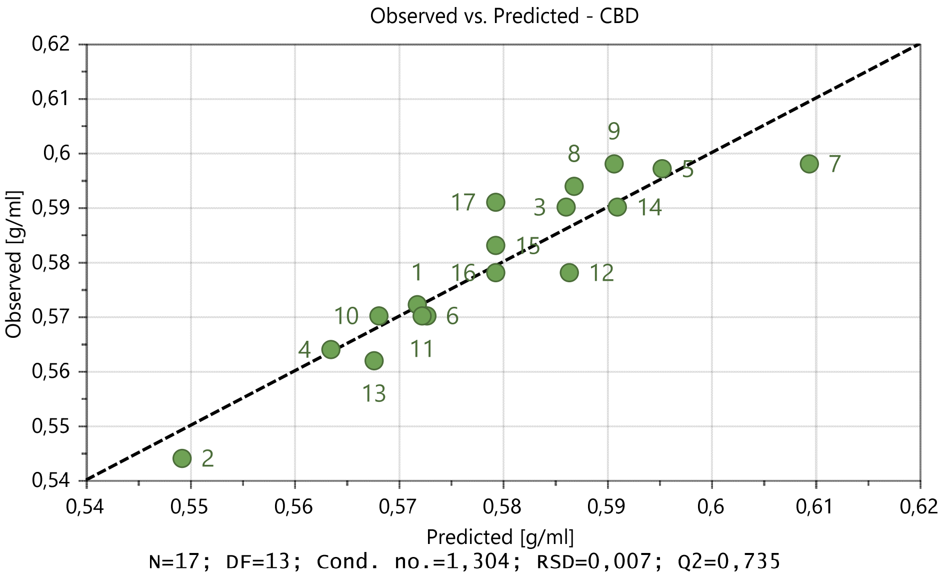

Figure S23. Comparison between observed and predicted results.

B.2 Summary of Fit: FRI

Data Transformation: -

Model Equation:

$$FRI = 0.803244 + 0.0077786 * THR + 2.41807 * 10^{-5} * HUM - 1.56664 * 10^{-5} * IMP - 0.000154715 * THR^2 - 1.02778 * 10^{-7} * HUM * IMP$$

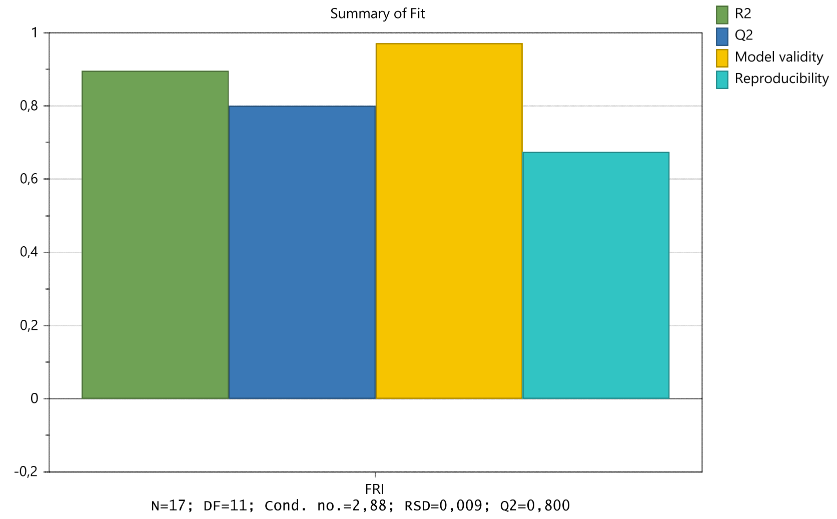

Figure S24. Summary of fit including R^2, Q^2, Model validity and Reproducibility.

Table S16. Fit statistics regarding Q^2, R^2 and R^2 adjusted.

Fit Statistics	
Q^2	0.800
R^2	0.896
R^2 adjusted	0.848

Table S17. Significance of the obtained model.

	Model	Lack of Fit
F-Value	18.8848	0.347358
P-Value	0.000	0.892
Significant?	yes	no

<u>Residuals Normal Probability</u>

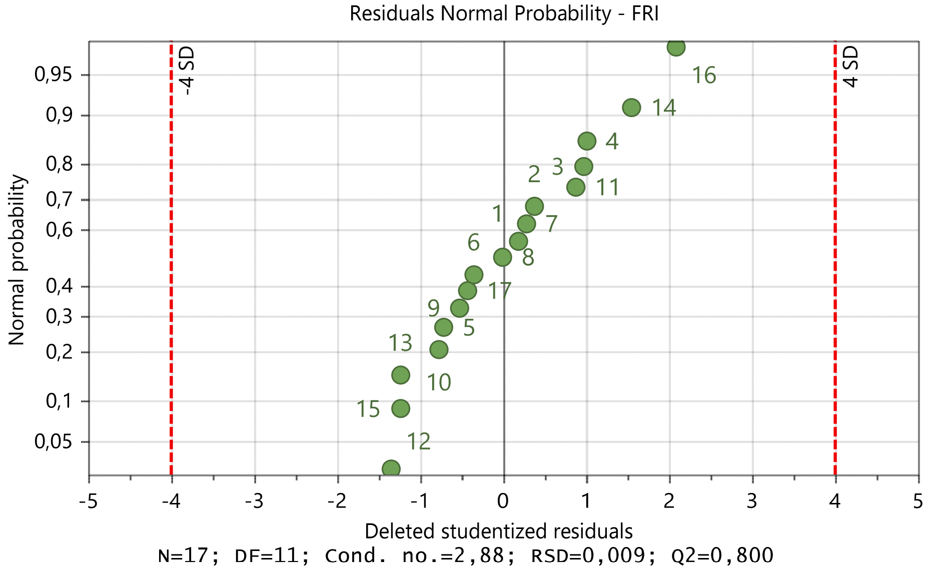

Figure S25. Residuals Normal Probability plot.

Observed vs Predicted

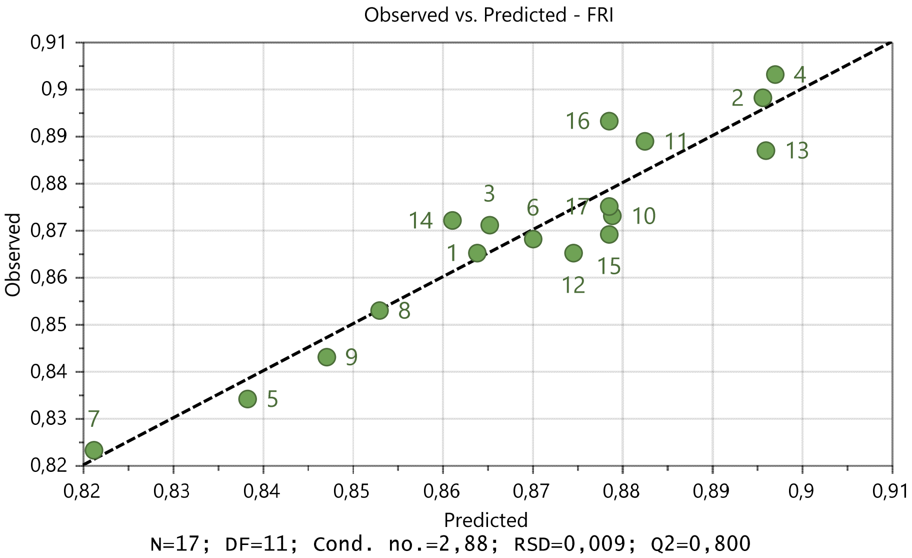

Figure S26. Comparison between observed and predicted results.

B.3 Summary of Fit: D10

Data Transformation: -

Model Equation:

$$d_{10} = 41.7377 - 0.839812 * THR - 0.00661847 * HUM - 0.00185889 * IMP + 0.0118172 * IMP^2 + 0.000291875 * THR * HUM + 1.38611 * 10^{-5} * HUM * IMP$$

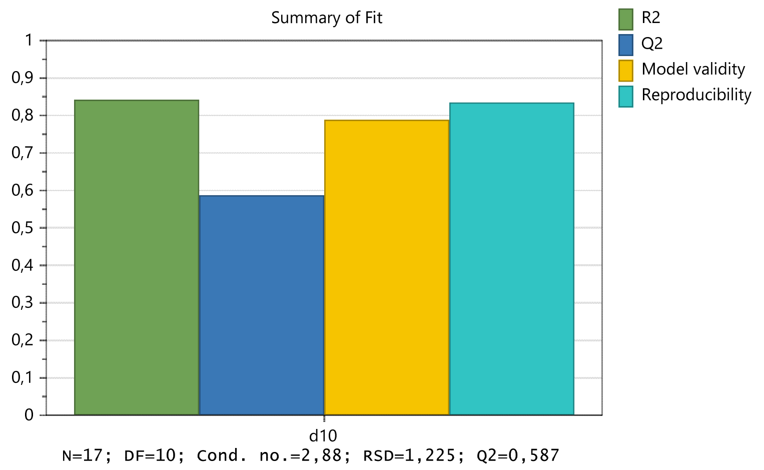

Figure S27. Summary of fit including R^2, Q^2, Model validity and Reproducibility.

Table S18. Fit statistics regarding Q^2, R^2 and R^2 adjusted.

Fit Statistics	
Q^2	0.587
R^2	0.842
R^2 adjusted	0.747

Table S19. Significance of the obtained model.

	Model	Lack of Fit
F-Value	8.89124	1.65981
P-Value	0.002	0.429
Significant?	yes	no

Residuals Normal Probability

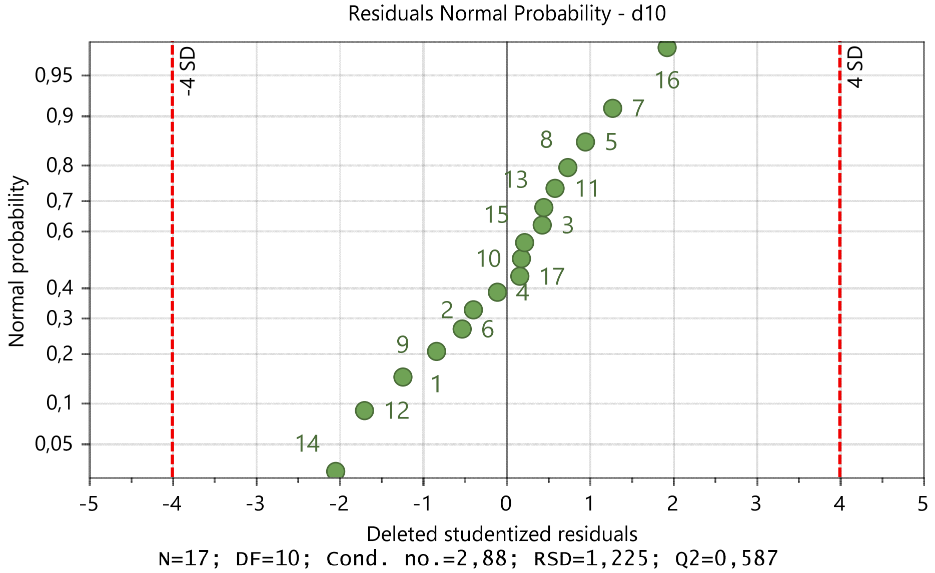

Figure S28. Residuals Normal Probability plot.

Observed vs Predicted

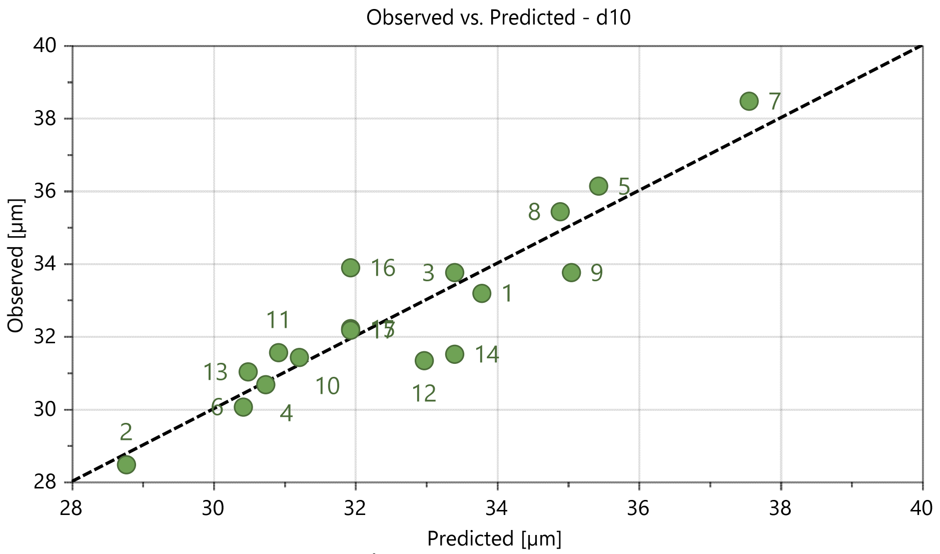

N=17; DF=10; Cond. no.=2,88; RSD=1,225; Q2=0,587

Figure S29. Comparison between observed and predicted results.

B.4 Summary of Fit: Compressibility

Data Transformation: -

Model Equation:

$$Compressibility = 0.584809 - 0.00112 * THR + 7.04581 * 10^{-5} * HUM + 0.000102111 * IMP - 9.16666 * 10^{-8} * HUM * IMP$$

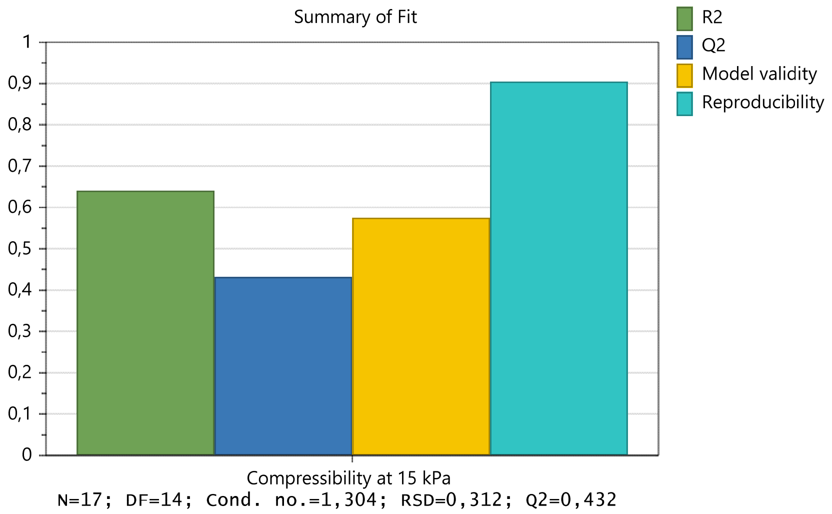

Figure S30. Summary of fit including R^2, Q^2, Model validity and Reproducibility.

Table S20. Fit statistics regarding Q^2, R^2 and R^2 adjusted.

Fit Statistics	
Q^2	0.432
R^2	0.641
R^2 adjusted	0.590

Table S21. Significance of the obtained model.

	Model	Lack of Fit
F-Value	12.4954	4.85968
P-Value	0.001	0.183
Significant?	yes	no

Residuals Normal Probability

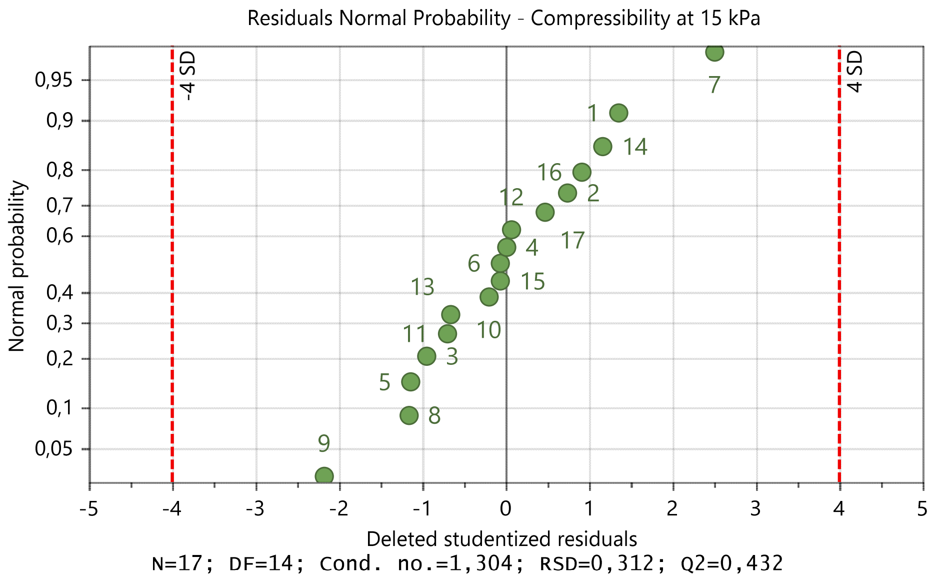

Figure S31. Residuals Normal Probability plot.

Observed vs Predicted

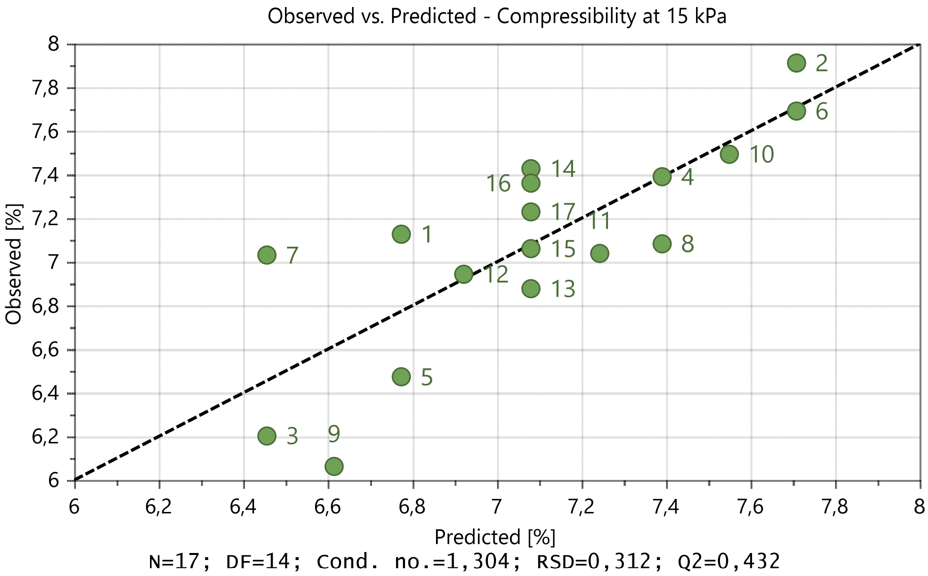

Figure S32. Comparison between observed and predicted results.

C. Summary of Fit: Tablet Press Parameters

C.1 Summary of Fit: Fill Depth

Data Transformation: -

Model Equation:

$$FD = 9.63996 + 0.0701824 * THR + 0.000317503 * HUM - 0.000564445 * IMP - 0.000811434 * THR^2 - 2.18752 * 10^{-5} * THR * HUM$$

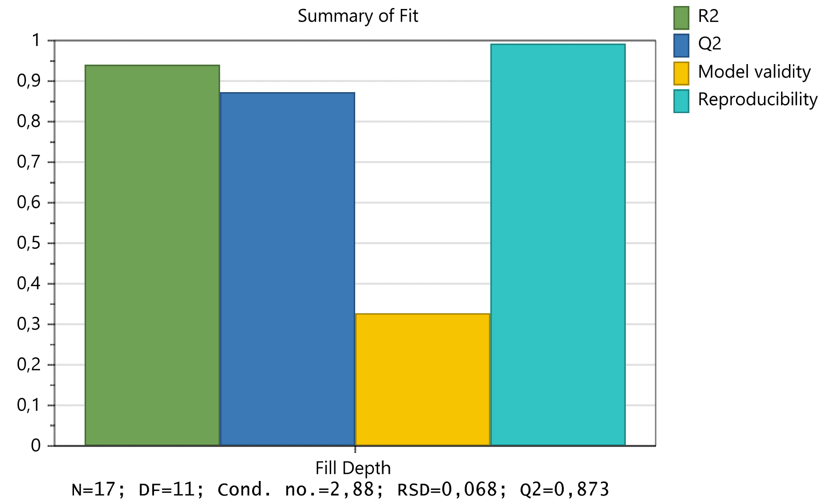

Figure S33. Summary of fit including R^2, Q^2, Model validity and Reproducibility.

Table S22. Fit statistics regarding Q^2, R^2 and R^2 adjusted.

Fit Statistics	
Q^2	0.873
R^2	0.941
R^2 adjusted	0.914

Table S23. Significance of the obtained model.

	Model	Lack of Fit
F-Value	34.8267	14.0723
P-Value	0.000	0.068
Significant?	yes	no

<u>Residuals Normal Probability</u>

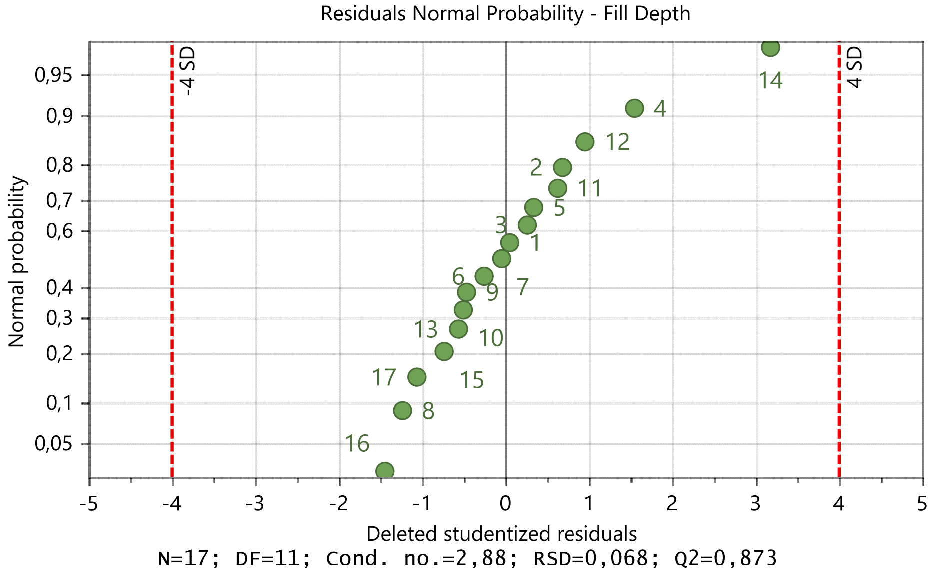

Figure S34. Residuals Normal Probability plot.

<u>Observed vs Predicted</u>

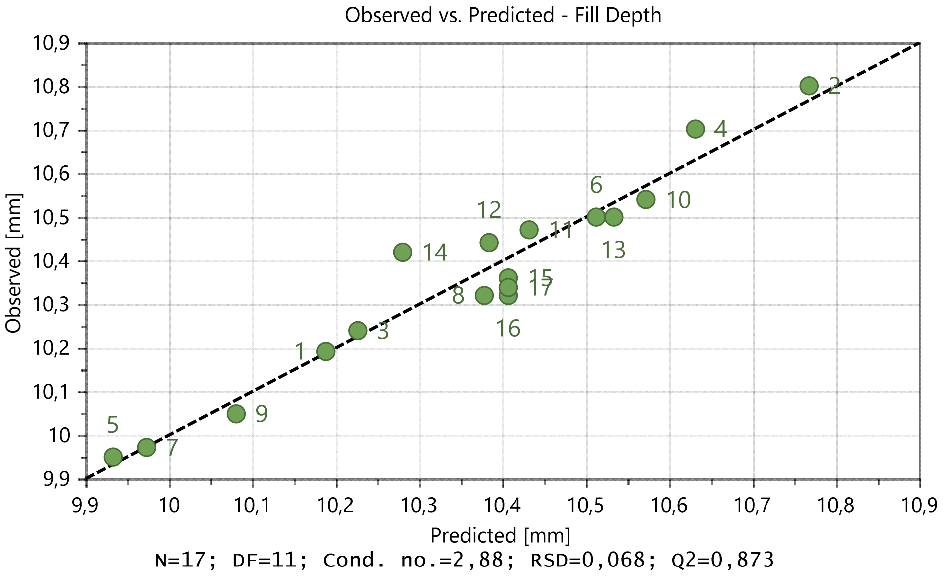

Figure S35. Comparison between observed and predicted results.

C.2 Summary of Fit: Bottom Main Compression Height

Data Transformation: -

Model Equation:

$$BCH = 5.11499 + 0.00797587 * THR + 8.49982 * 10^{-5} * HUM - 0.000414171 * IMP - 0.000187731 * THR^2 + 4.19285 * 10^{-7} * IMP^2 + 6.6667 * 10^{-6} * THR * IMP$$

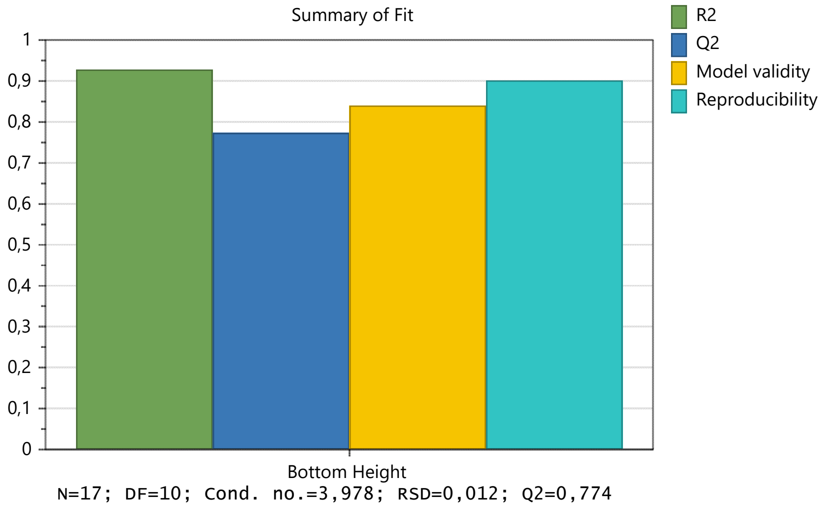

Figure S36. Summary of fit including R^2, Q^2, Model validity and Reproducibility.

Table S24. Fit statistics regarding Q^2, R^2 and R^2 adjusted.

Fit Statistics	
Q^2	0.774
R^2	0.928
R^2 adjusted	0.885

Table S25. Significance of the obtained model.

	Model	Lack of Fit
F-Value	21.4859	1.21091
P-Value	0.000	0.528
Significant?	yes	no

Residuals Normal Probability

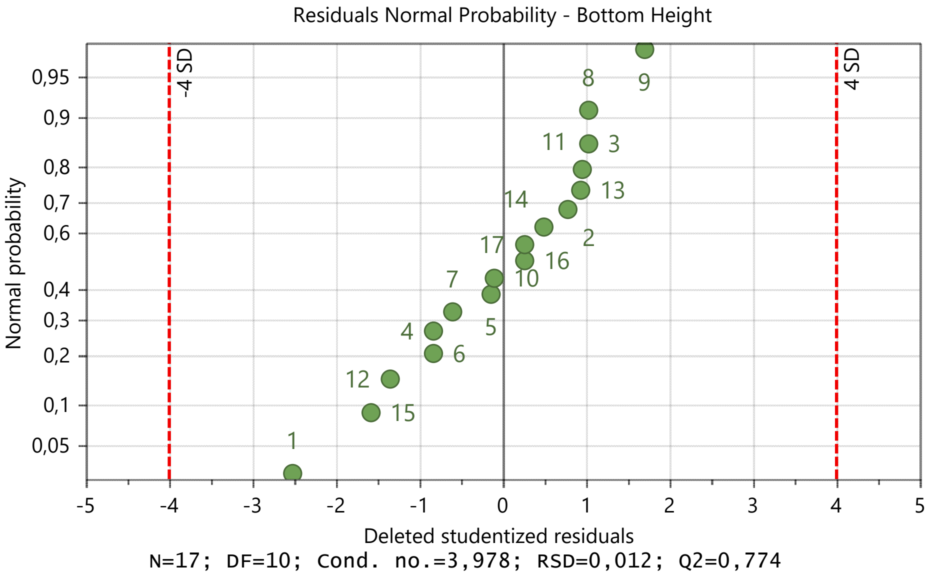

Figure S37. Residuals Normal Probability plot.

Observed vs Predicted

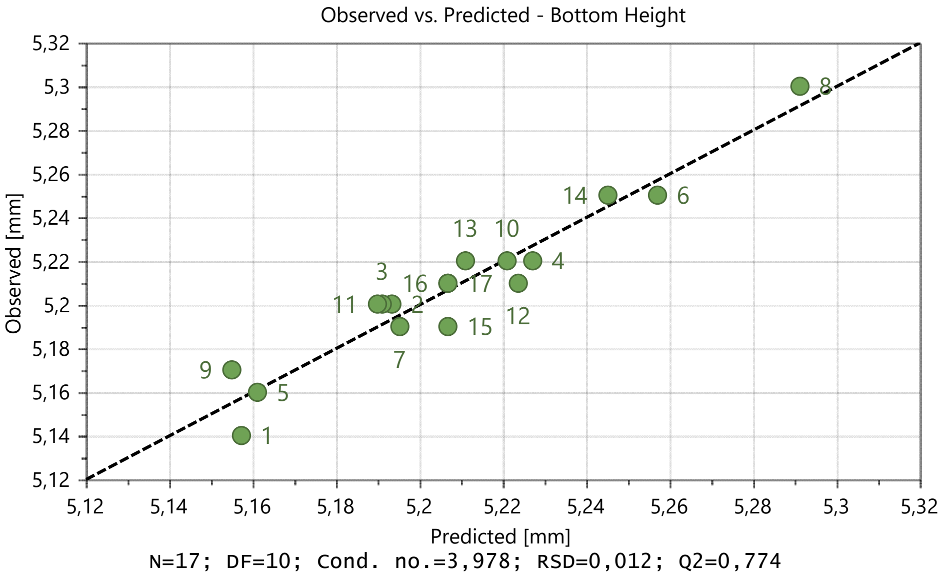

Figure S38. Comparison between observed and predicted results.

C.3 Summary of Fit: Ejection Force

Data Transformation: -

Model Equation:

$$EF = 0.0260715 + 0.00774714 * THR - 1.2 * 10^{-5} * IMP - 0.000184428 * THR^2$$

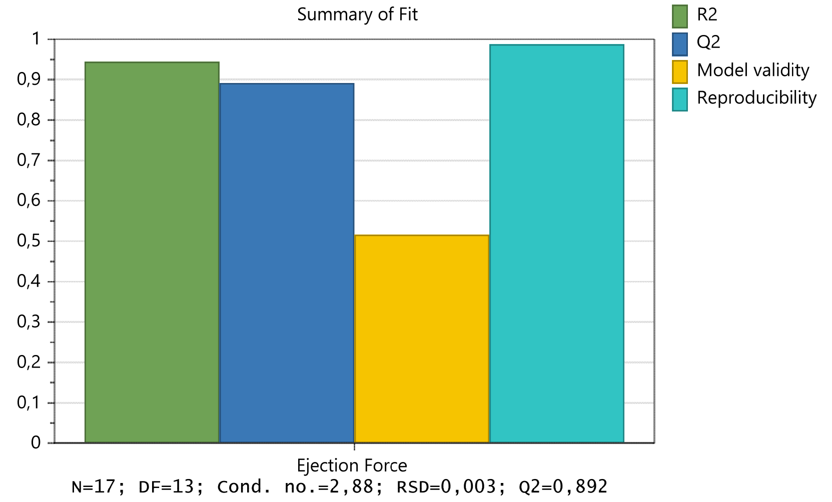

Figure S39. Summary of fit including R^2, Q^2, Model validity and Reproducibility.

Table S26. Fit statistics regarding Q^2, R^2 and R^2 adjusted.

Fit Statistics	
Q^2	0.892
R^2	0.944
R^2 adjusted	0.931

Table S27. Significance of the obtained model.

	Model	Lack of Fit
F-Value	73.3341	6.30616
P-Value	0.000	0.145
Significant?	yes	no

Residuals Normal Probability

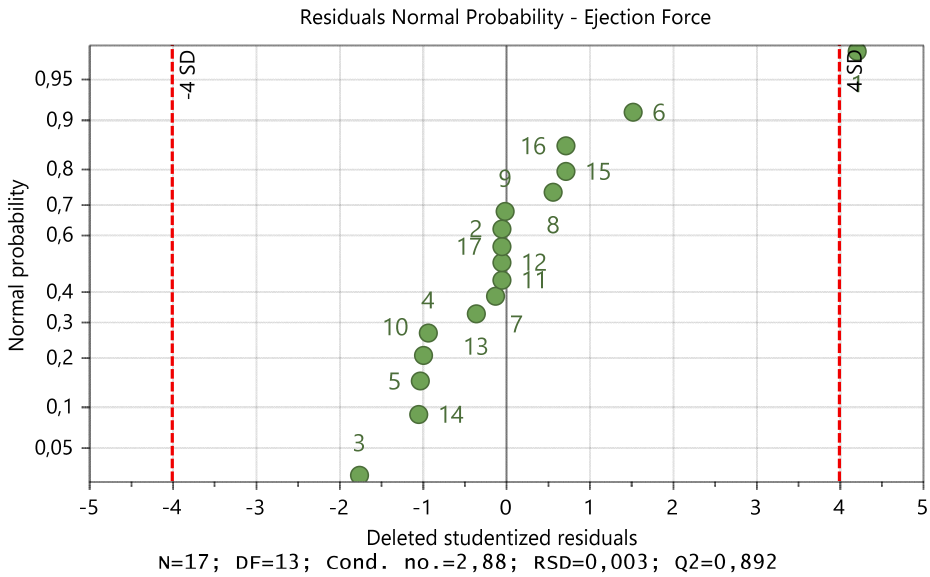

Figure S40. Residuals Normal Probability plot.

Observed vs Predicted

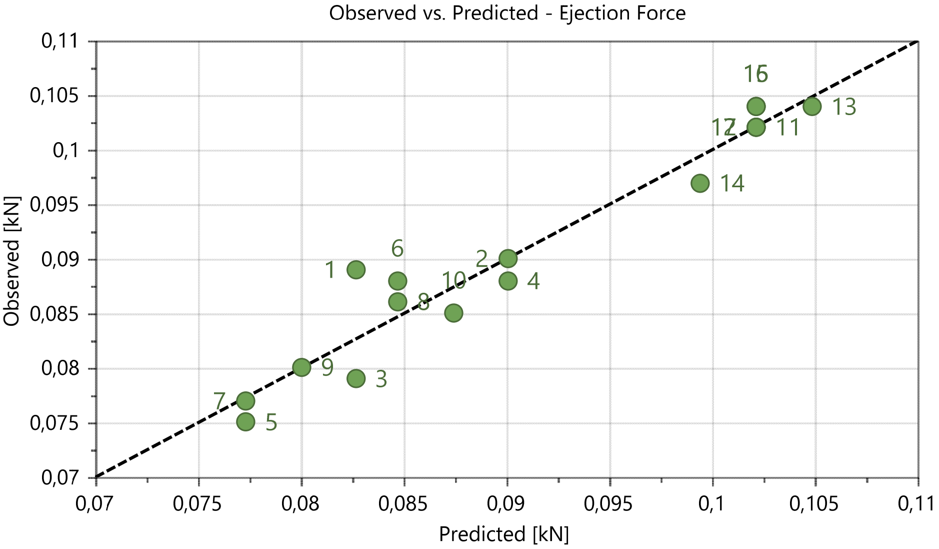

N=17; DF=13; Cond. no.=2,88; RSD=0,003; Q2=0,892

Figure S41. Comparison between observed and predicted results.

D. Summary of Fit: Tablet Properties

D.1 Summary of Fit: Tensile Strength at 275 MPa

Data Transformation: logarithmic

Model Equation:

$$Log_{10}(TS) = 0.364822 + 0.017798 * THR - 0.000162112 * HUM - 0.00030635 * IMP - 0.000204041 * THR^2 - 3.32993 * 10^{-6} * THR * HUM - 9.37246 * 10^{-6} * THR * IMP + 3.0309 * 10^{-7} * HUM * IMP$$

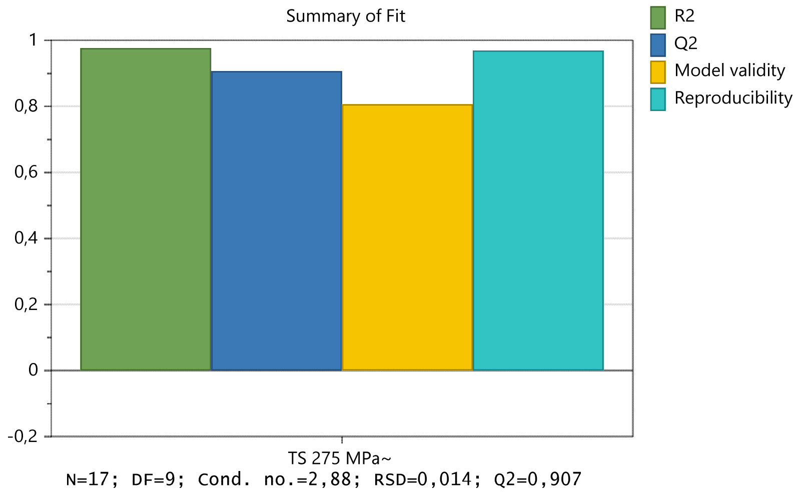

Figure S42. Summary of fit including R^2, Q^2, Model validity and Reproducibility.

Table S28. Fit statistics regarding Q^2, R^2 and R^2 adjusted.

Fit Statistics	
Q^2	0.907
R^2	0.976
R^2 adjusted	0.958

Table S29. Significance of the obtained model.

	Model	Lack of Fit
F-Value	52.7115	1.47628
P-Value	0.000	0.462
Significant?	yes	no

Residuals Normal Probability

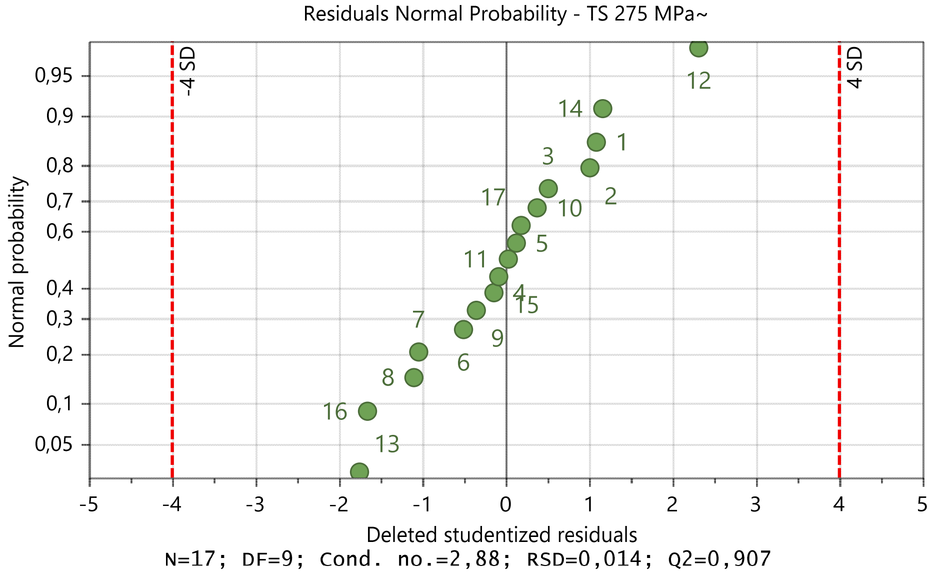

Figure S43. Residuals Normal Probability plot.

Observed vs Predicted

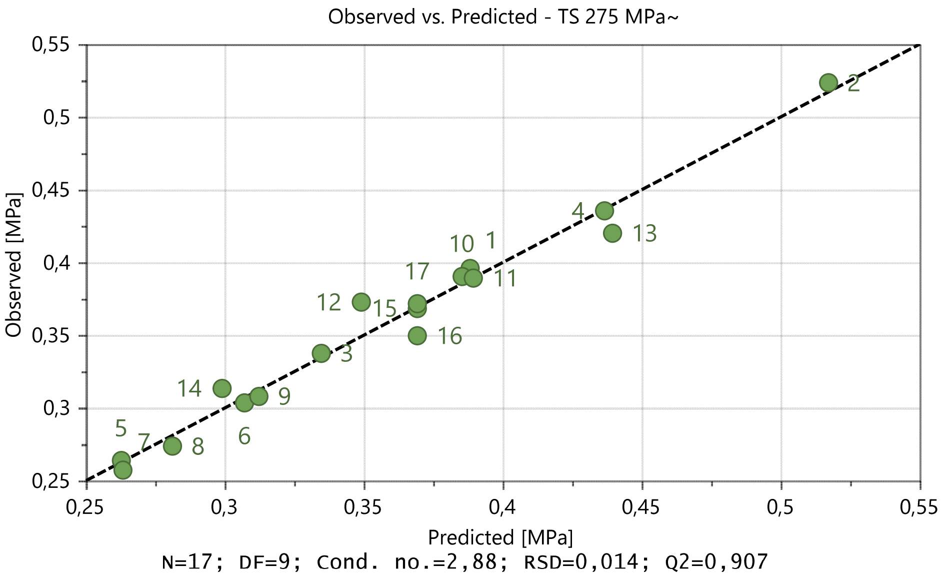

Figure S44. Comparison between observed and predicted results.

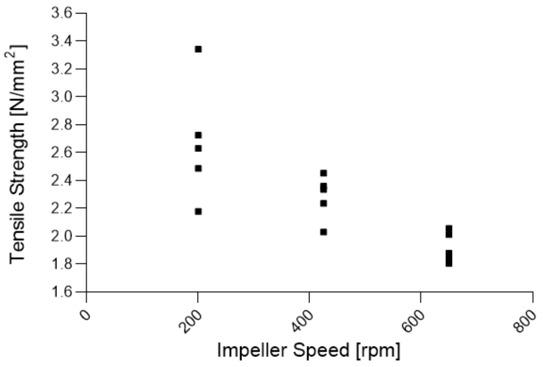

Figure S45. TS as function of impeller speed. Data transformation was conducted since variability is not constant.

D.2 Summary of Fit: Tablet Weight at 275 MPa

Data Transformation: logarithmic

Missing model validity due to minimal variability in Tablet weight at the three replicates – two values are identical (597.97 mg / 597.97 mg/ 598.34 mg).

Model Equation:

$$Log_{10}(TW) = 604.751 + 0.245558 * THR + 0.0126548 * HUM - 0.0934296 * IMP - 0.0142736 * THR^2 + 9.65444 * 10^{-5} * IMP^2 + 0.00117056 * THR * IMP$$

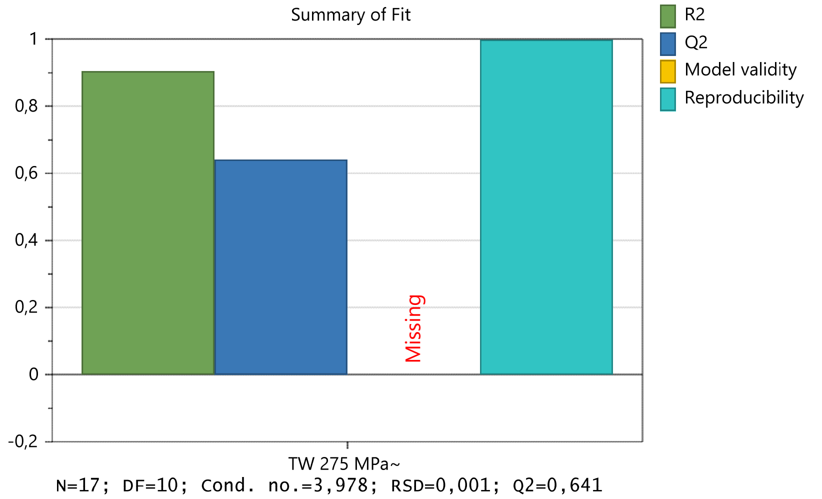

Figure S46. Summary of fit including R^2, Q^2, Model validity and Reproducibility.

Table S30. Fit statistics regarding Q^2, R^2 and R^2 adjusted.

Fit Statistics	
Q^2	0.641
R^2	0.904
R^2 adjusted	0.847

Table S31. Significance of the obtained model.

	Model	Lack of Fit
F-Value	15.723	--
P-Value	0.000	--
Significant?	yes	--

<u>Residuals Normal Probability</u>

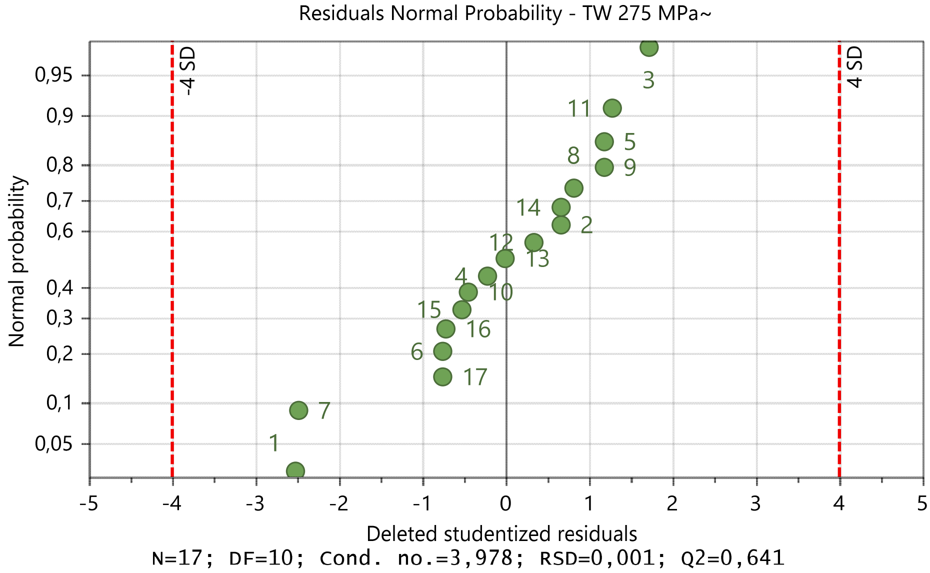

Figure S47. Residuals Normal Probability plot.

Observed vs Predicted

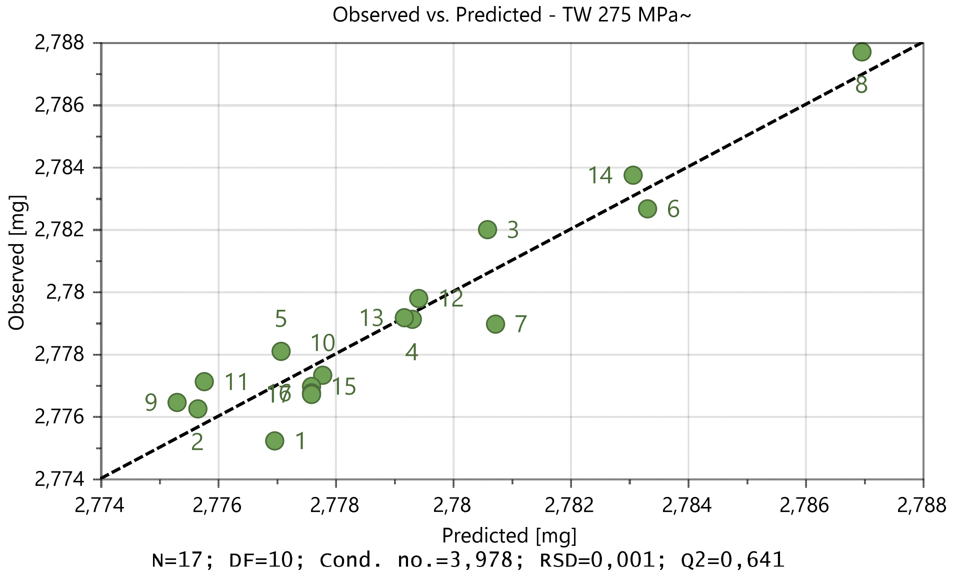

Figure S48. Comparison between observed and predicted results.

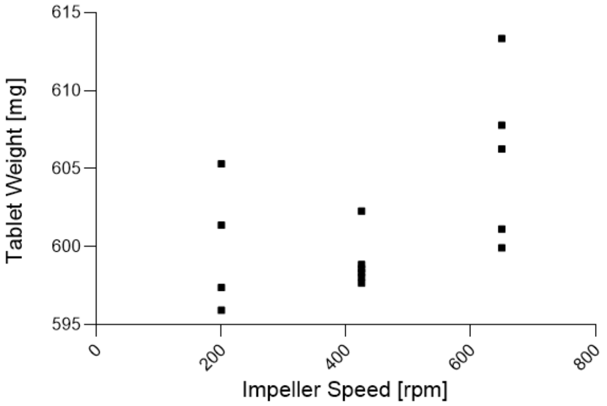

Figure S49. TW as function of impeller speed. Data transformation was conducted since variability is not constant.

D.3 Summary of Fit: Tablet Thickness at 275 MPa

Data Transformation: logarithmic

Model Equation:

$$Log_{10}(TT) = 0.680735 + 0.000321726 * THR + 1.6724 * 10^{-5} * HUM - 4.24101 * 10^{-5} * IMP - 1.32394 * 10^{-5} * THR^2 + 5.31267 * 10^{-8} * IMP^2 + 1.0325 * 10^{-6} * THR * IMP - 2.25652 * 10^{-8} * HUM * IMP$$

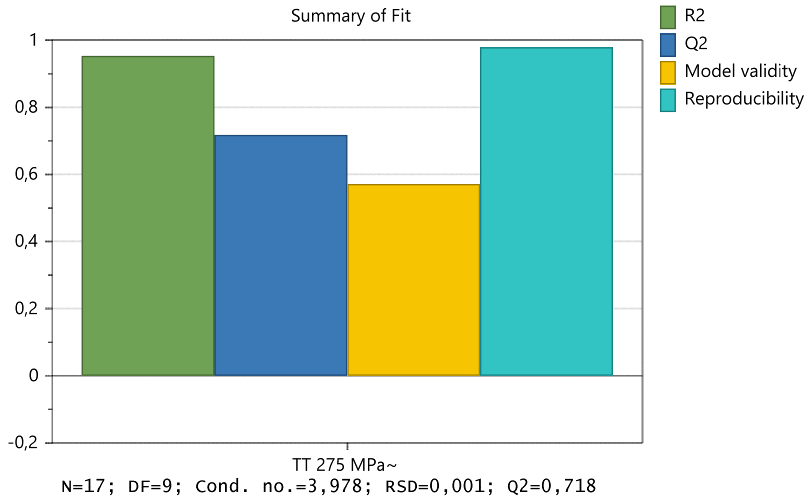

Figure S50. Summary of fit including R^2, Q^2, Model validity and Reproducibility.

Table S32. Fit statistics regarding Q^2, R^2 and R^2 adjusted.

Fit Statistics	
Q^2	0.718
R^2	0.953
R^2 adjusted	0.917

Table S33. Significance of the obtained model.

	Model	Lack of Fit
F-Value	26.1235	4.88732
P-Value	0.000	0.18
Significant?	yes	no

Residuals Normal Probability

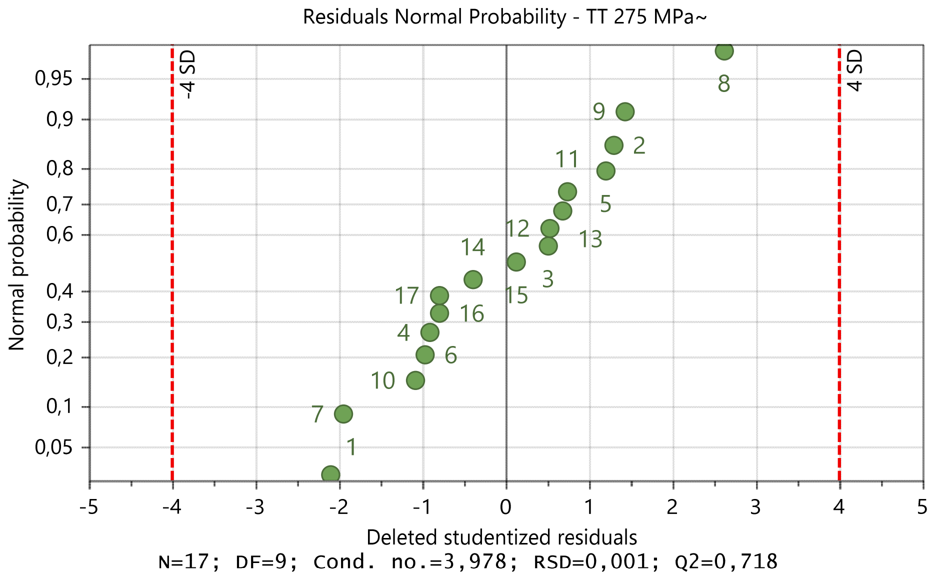

Figure S51. Residuals Normal Probability plot.

<u>Observed vs Predicted</u>

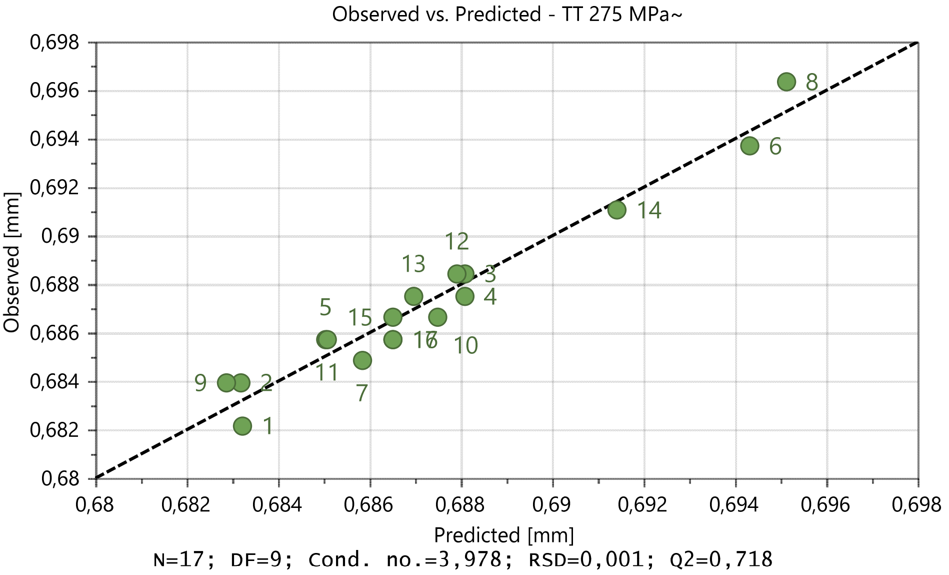

Figure S52. Comparison between observed and predicted results.

Data transformation since variability is not constant

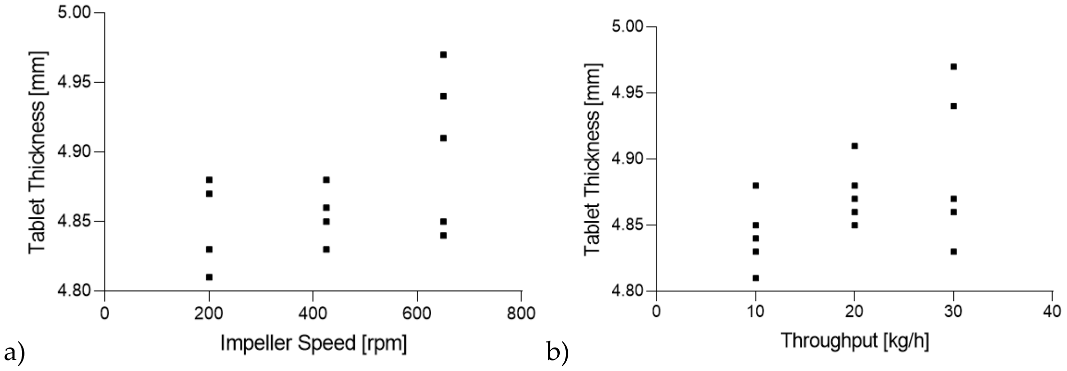

Figure S53. a) TT as function of impeller speed. b) TT as function of throughput. Data transformation was conducted since variability is not constant.

E. Additional Demonstration of HUM StDev

To demonstrate the impact of impeller speed, Figure S54 shows two PI Vision screenshots of phase 13 and 14 of the DoE. In both runs HUM and throughput were 400 g and 30 kg/h respectively. Impeller speed was adjusted at 200 rpm in **Figure S54** a) and 650 rpm in **Figure S54** b). The y-axis of both figures shares the same values for HUM PV and SP, whereas the numbers regarding EV differ due to the phases' vast discrepancies. In Figure S54 a) consistent HUM and EV trajectories are shown.

In **Figure S*54*** b) it can be observed that at the beginning of the process (after a process stop) the HUM increased the most. In general, high impeller speeds push the powder at the walls and in the upper region of the mixer. The amount of powder in the area close to the exit valve decreases, why the exit valve has to increase the open width to ensure a consistent outflow out of the mixer.[1]

Since the impeller speed was too high at these settings, hardly powder could leave the CMT and built up. Simultaneously, the exit valve opening width increased to extreme values to compensate for the accumulation until it overshoots. To balance this overshoot, the EV open width decreased, undershoot and HUM rose again. This procedure went on until it was balanced out. This way, it only slowly adjusted to the HUM SP causing fluctuations.

It is essential to avoid such process states to enable a steady-state condition quickly. For example, that is vital to adjust the fill depth to meet the weight specifications. Since HUM is used to determine the TBP, highly fluctuating blend conditions resulted in discrepancies regarding the blend's powder attributes and affected the tablet properties. Hence, fill depth could not be adjusted among others since the density of the powder frequently changed due to fluctuations causing variability in tablet weight.

a) phase 13

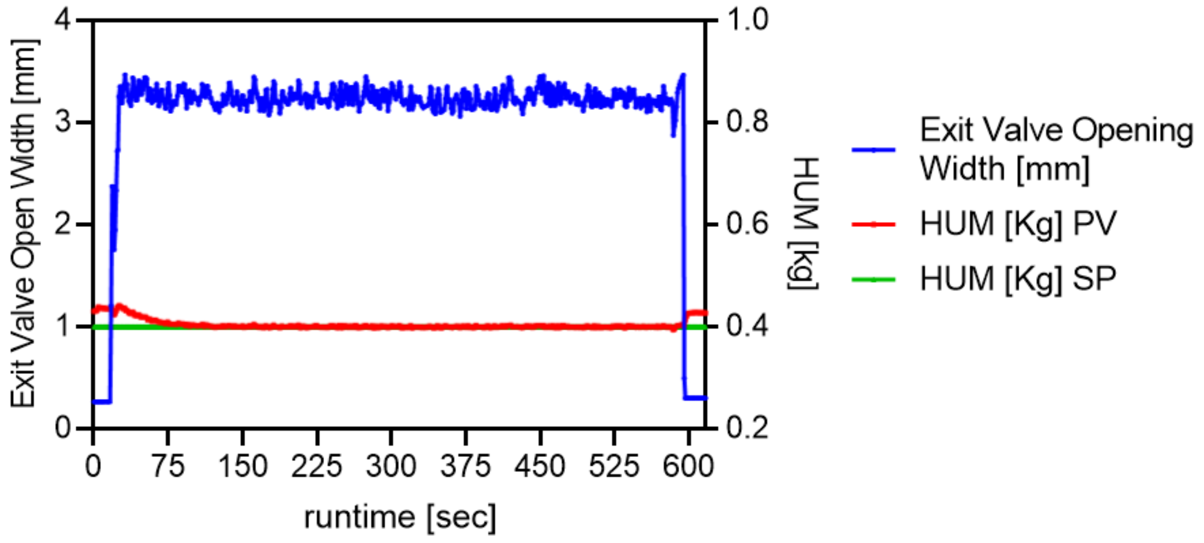

b) phase 14

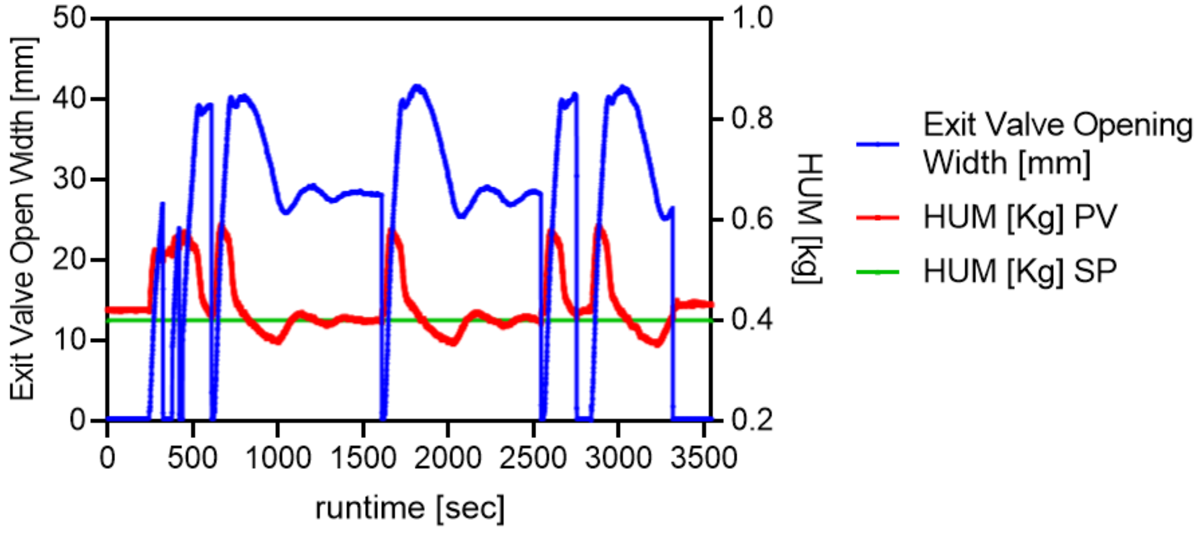

Figure S54. a) HUM set point = 0.4 kg (green), HUM process value (blue) and corresponding EV (red) at 200 rpm impeller speed during steady state – approx. 10 min. b) HUM set point = 0.4 kg (green), HUM process value (blue) and corresponding EV (red) at 650 rpm impeller speed for approx. 1 h.

F. Raw Material Attributes

Table S34. Density and particle size distribution of the individual raw materials.

	Bulk Density [g/ml]	Tapped Density [g/ml]	Hausner Ratio	Carr Index	D10 [µm]	D50 [µm]	D90 [µm]
Microcrystalline Cellulose	0.35	0.47	1.36	26.28	40.53	113.35	222.04
Sodium Saccharin Monohydrate	0.77	0.89	1.15	13.40	27.46	208.94	374.48
Di-Claclium Phosphate	0.76	0.85	1.12	10.65	38.58	109.11	213.9
Sodium Starch Glycolate	0.73	0.87	1.18	15.57	26.07	50.03	74.18
Magnesiumstearate	0.17	0.24	1.43	30.31	12.12	39.05	289.52

G. Ejection Force

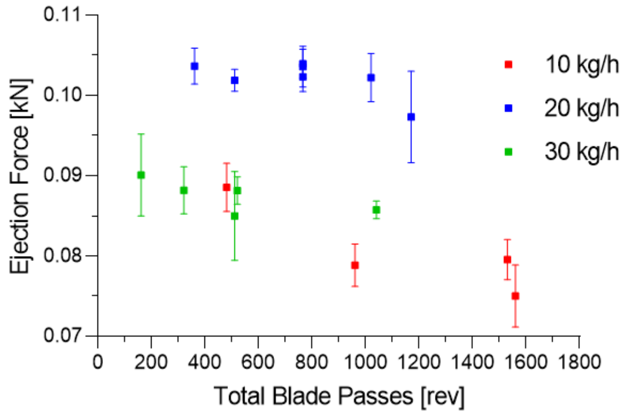

Figure S55. Ejection force as function of TBP.

Regarding TBP and ejection force, curiously, the values at 20 kg/h were higher than at 10 and 30 kg/h. Considered individually, the correlation between TBP and the values at 20 kg/h are not significant (-0,658 p=0,108). Contrary, values at 10 kg/h and 30 kg/h combined showed a highly significant and strong correlation (-0.911 p=0.0002), where higher TBP and higher lubrication respectively resulted in lower Ejection Forces. Why only the data at 20 kg/h were higher could not be explained by the available data. The same phenomena could be observed at ejection force in dependency of particle size (d_{10}), where only data regarding 10 kg/h & 30 kg/h showed a correlation (-0.789 p=0.007).[20 kg/h (0,313 p=0,494)]

The influence of particle size on ejection force can be traced back again on the lubrication described previously.

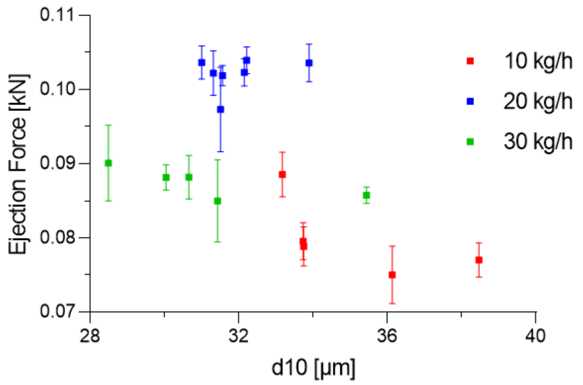

Figure S56. Ejection force as function of particle size (d_{10}).

H. Correlation Matrix

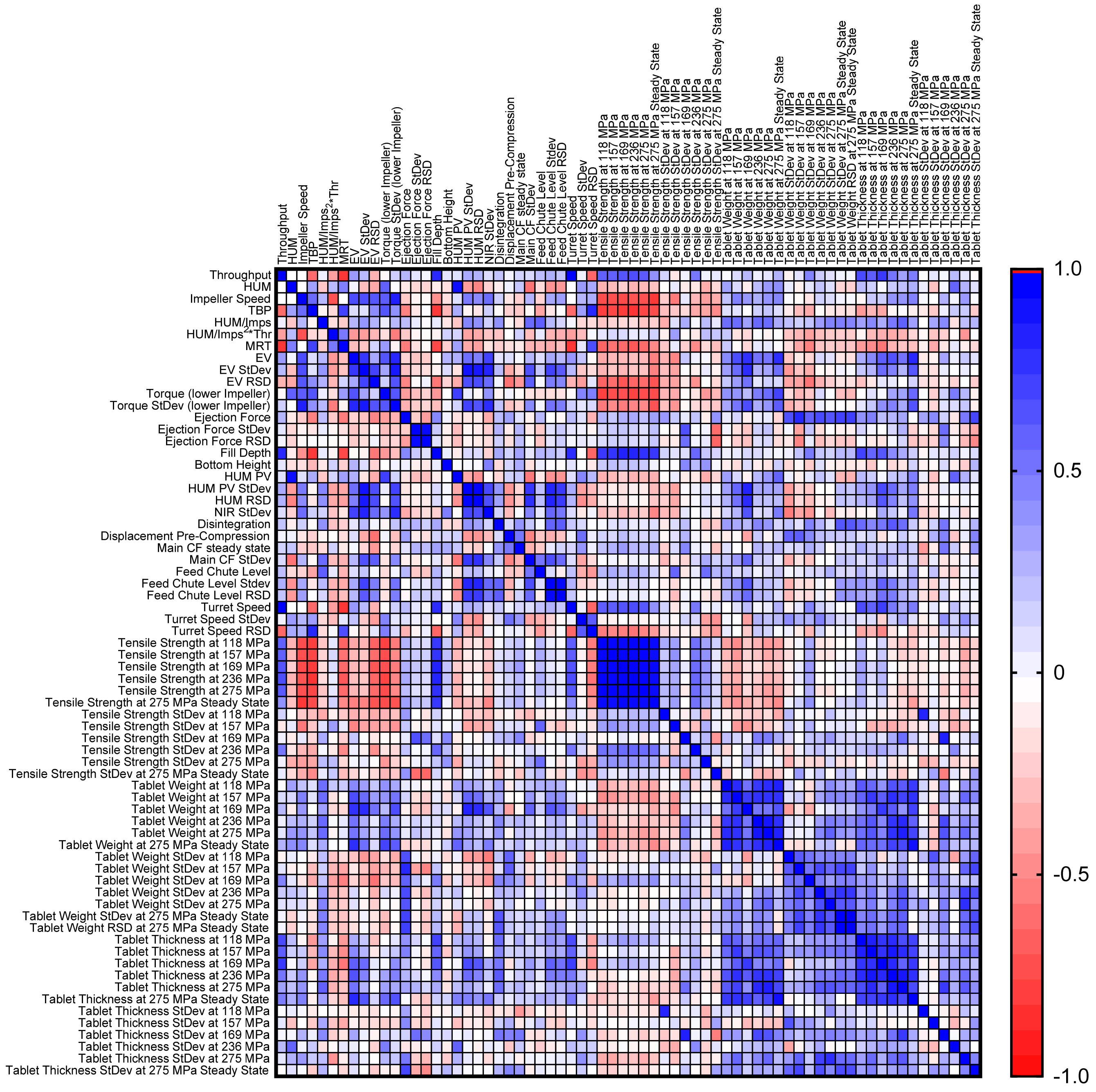

Figure S57. Correlation matrix of the input parameters and responses considered in this work.

I. References

(1) Toson, P.; Siegmann, E.; Trogrlic, M.; Kureck, H.; Khinast, J.; Jajcevic, D.; Doshi, P.; Blackwood, D.; Bonnassieux, A.; Daugherity, P. D.; am Ende, M. T. Detailed Modeling and Process Design of an Advanced Continuous Powder Mixer. *International Journal of Pharmaceutics* **2018**, *552* (1–2), 288–300. https://doi.org/10.1016/j.ijpharm.2018.09.032.
